# Activity-Related Conformational Changes in d,d-Carboxypeptidases Revealed by *In Vivo* Periplasmic Förster Resonance Energy Transfer Assay in *Escherichia coli*

**DOI:** 10.1128/mBio.01089-17

**Published:** 2017-09-12

**Authors:** Nils Y. Meiresonne, René van der Ploeg, Mark A. Hink, Tanneke den Blaauwen

**Affiliations:** a Bacterial Cell Biology and Physiology, Swammerdam Institute for Life Sciences, University of Amsterdam, Amsterdam, The Netherlands; b Molecular Cytology and van Leeuwenhoek Centre for Advanced Microscopy, Swammerdam Institute for Life Sciences, University of Amsterdam, Amsterdam, The Netherlands; Max Planck Institute for Terrestrial Microbiology

**Keywords:** FRET, PBP5, PBP6a, PBP6b, antibiotics, mCherry, mNeonGreen, periplasm, protein interactions

## Abstract

One of the mechanisms of β-lactam antibiotic resistance requires the activity of d,d-carboxypeptidases (d,d-CPases) involved in peptidoglycan (PG) synthesis, making them putative targets for new antibiotic development. The activity of PG-synthesizing enzymes is often correlated with their association with other proteins. The PG layer is maintained in the periplasm between the two membranes of the Gram-negative cell envelope. Because no methods existed to detect *in vivo* interactions in this compartment, we have developed and validated a Förster resonance energy transfer assay. Using the fluorescent-protein donor-acceptor pair mNeonGreen-mCherry, periplasmic protein interactions were detected in fixed and in living bacteria, in single samples or in plate reader 96-well format. We show that the d,d-CPases PBP5, PBP6a, and PBP6b of *Escherichia coli* change dimer conformation between resting and active states. Complementation studies and changes in localization suggest that these d,d-CPases are not redundant but that their balanced activity is required for robust PG synthesis.

## IMPORTANCE

The periplasmic space between the outer and the inner membrane of Gram-negative bacteria contains many essential regulatory, transport, and cell wall-synthesizing and -hydrolyzing proteins. To date, no assay is available to determine protein interactions in this compartment. We have developed a periplasmic protein interaction assay for living and fixed bacteria in single samples or 96-well-plate format. Using this assay, we were able to demonstrate conformation changes related to the activity of proteins that could not have been detected by any other living-cell method available. The assay uniquely expands our toolbox for antibiotic screening and mode-of-action studies.

## INTRODUCTION

The envelope of Gram-negative bacteria consists of an outer membrane (OM) that is permeable to small molecules and an inner membrane (IM). The space between these membranes is termed the periplasm, a distinct compartment in which the peptidoglycan (PG) layer is maintained. The PG layer is built from alternating *N*-acetylglucosamine and *N*-acetylmuramyl-pentapeptide disaccharide units that form a covalently closed network of glycan strands interconnected by peptide bridges that is able to withstand the internal turgor pressure of the cells (1, 2). It determines the shape of the cells and offers the rigidity cells need to function and survive under changing conditions. The PG layer is under constant reconstruction by penicillin binding proteins (PBPs) and other proteins that expand, break down, and modify it to allow cells to grow and divide while preventing lysis. PBPs are classified by molecular mass and function (3, 4). The bifunctional class A PBP1a and PBP1b polymerize the glycan strands of PG by glycosyltransferase (GTase) activity and cross-link their peptide side chains by transpeptidation (d,d-TPase). The monofunctional class B PBP2 and PBP3 exhibit essential d,d-TPase activities associated with elongation and division, respectively (5). Class C PBPs have PG-modifying activities and are further classified by their ability to hydrolyze the peptide bond between the peptide side chains and the PG glycan strands through endopeptidase (d,d-EPase) activity (PBP4, PBP4b, and PBP7) or carboxypeptidase (d,d-CPase) activity (PBP5, PBP6a, and PBP6b) that cleaves off the pentapeptide terminal residue d-Ala. Inhibition of PBPs kills bacteria very effectively, as demonstrated by the success of β-lactam antibiotics. β-Lactams structurally resemble the d-Ala-d-Ala substrate moiety of PG precursors and form an ester bond with the active site of d,d-TPases, rendering them inactive (6). However, this antibiotic therapy is rapidly becoming ineffective due to the development of resistance mechanisms (7).

Resistance against β-lactams is achieved by the production of hydrolyzing β-lactamases (8) or drug efflux pumps (9), adaptation of membrane permeability (10), or replacement of the standard 4-3 cross-links with 3-3 cross-links (11). In *Escherichia coli*, about 3% or 10% of PG cross-links are of the 3-3 type when grown under exponential or stationary growth conditions, respectively (12), but 3-3 cross-links can also fully replace 4-3 cross-links when d,d-TPases are bypassed during β-lactam resistance (13). The unusual 3-3 cross-links are catalyzed by l,d-transpeptidases (l,d-TPases), which are structurally unrelated to PBPs and are not targeted by most β-lactams (12, 14). l,d-TPase-mediated cross-linking and β-lactam resistance in *E. coli* are dependent on GTases and the otherwise dispensable d,d-CPase PBP5 (*dacA*), which has a natural resistance to β-lactams (13). By removing the last d-Ala of the pentapeptide, PBP5 provides the tetrapeptide substrate for the l,d-TPase. Pathogenic bacteria from broadly different lineages like *Clostridium difficile* (15), *Enterococcus faecium* (11), and *Mycobacterium tuberculosis* (16) all show 3-3 cross-linking related to β-lactam resistance. Cases in which large percentages of 3-3 PG cross-links confer β-lactam resistance would thus benefit if d,d-CPase activity could be coinhibited to prevent l,d-TPase activity.

d,d-CPase inhibitors have been developed using PBP5 as a model enzyme by generating specific substrates like cyclic peptides or boronates (17, 18). Although single deletions of d,d-CPases elicit only mild phenotypes in *E. coli*, the additional deletion of multiple enzymes results in cells that are unable to maintain their morphology, suggesting at least partly complementing functions (19–21). This makes it challenging to distinguish between individual d,d-CPase functions. For instance, d,d-CPase activity only of PBP5 was found to confer l,d-TPase-mediated β-lactam resistance, as the deletion of *dacA* resulted in decreased formation of resistant mutants, which was not observed for single PBP6a (*dacC*) or PBP6b (*dacD*) deletions (13). The remaining d,d-CPase activity of PBP5 in the latter mutants may have been sufficient for l,d-TPase activity, whereas PBP6a and PBP6b activities in the *ΔdacA* strain were clearly insufficient under the conditions tested. Apart from maintaining cell morphology, d,d-CPases play additional roles in β-lactam resistance (21–23). PBP6b but not PBP6a overexpression partially restored β-lactam resistance in Δ*dacAD* and Δ*dacAC* strains (21). And yet, PBP6a variants appear to aid in the antibiotic resistance of clinically isolated *E. coli* strains (23) and, interestingly, PBP6b is required for the activity of certain β-lactamases (24). This underscores the need to consider a function for the presence of d,d-CPases, as well as for their activity.

PBP5, PBP6a, and PBP6b comprise 50% of all PBPs in the cell (21), and ribosomal profiling revealed that PBP5 is about 2 times more abundant than PBP6a, while PBP6b is hardly expressed under laboratory conditions (25). However, PBP6b is upregulated under low-pH conditions and can complement d,d-CPase activity in a Δ*dacABCD* strain (26). The conditions that require a functional PBP6a and the precise substrate specificity of this protein are unknown. *In vitro* experiments show that it initiates a preacylation complex with the small penicillin analog Bocillin FL relatively easily compared to PBP5, followed by a rather slow hydrolysis step. The binding and hydrolysis by PBP6a of the larger substrate Nα,Nε-diacetyl-Lys-d-Ala-d-Ala is weaker than that of PBP5 and not even detectable for the biologically more relevant PG substrate mimic l-Ala-γ-d-Glu-l-Lys-d-Ala-d-Ala (21, 27).

PBP5, PBP6a, and PBP6b are structurally highly similar and are translated as preproteins with N-terminal signal sequences that are cleaved off after transport to the periplasm through the Sec translocon (28). Upon folding in the periplasm, the proteins consist of globular active-site domains and stalklike domains attached to the IM by C-terminal amphipathic helices (20). PBP5 localizes laterally (i.e., in the cylindrical part of the envelope) and in a substrate-dependent manner to the midcell, while the active-site mutant PBP5^S44G^ (expressing a change of Ser to Gly at position 44) is absent from the midcell and the poles (29). PBP6a and PBP6b localize laterally in Δ*dacC* or Δ*dacD* strains, respectively, and also to the septal ring in the absence of PBP5, suggesting complementary functions (28). PBP6a localizes to the septal sites better than PBP6b, which does localize strongly when cell division is also blocked by the PBP3 inhibitor aztreonam (28). This is interesting, as PBP6b is considered a complementing factor in d,d-CPase-deficient strains while PBP6a is not, suggesting a localization hierarchy.

*In vivo* detection of d,d-CPase interactions would significantly aid the study of their activities and functions, as well as the specificity of d,d-CPase-targeting compounds under different conditions. Unfortunately, this is hampered by the absence of available methods to study protein interactions in the periplasm. Truly *in vivo* experiments should be done in the compartment where the proteins of interest reside and function. Commonly used cytosolic methods like bacterial two-hybrid (B2H) assays are not available for the periplasm (30, 31). Förster resonance energy transfer (FRET) is a method that can detect protein interactions in the cytosol directly, without the need for transcription of reporters, as for B2H (32–36), and is thus conceptually applicable to the periplasm. For FRET analysis of *in vivo* protein interactions, a donor and an acceptor fluorescent protein (FP) are used, each being fused to one of two proteins that are suspected to interact. Close proximity of the donor and acceptor FP will allow energy transfer, suggesting interaction of the assayed proteins. Because to our knowledge such an assay did not exist for the periplasm, we developed a FRET assay that works in this compartment. Well-known FRET pairs are cyan fluorescent protein-yellow fluorescent protein (YFP) and monomeric Kusabira orange (mKO)-mCherry (mCh) (32, 35–38). Whereas a wealth of FP color variants is available for the cytosol, only a few are able to fold, mature, and fluoresce in the oxidative environment of the periplasm (39–43). Of these functional FPs, superfolder green fluorescent protein (sfGFP) and mCh can be used as a FRET pair, but the translocation of sfGFP to the periplasm resulted in cytotoxic effects and loss of fluorescence, making it suboptimal for *in vivo* FRET assays. Here, we report for the first time that mNeonGreen (mNG) is able to fold and mature in the periplasm and can be used as a donor FP. We used the mNG-mCh FRET pair to develop the first periplasmic FRET assay. As a proof of concept, mCh fused to mNG was expressed in the periplasm and FRET was detected by spectral unmixing and confirmed by fluorescence lifetime-imaging microscopy (FLIM).

The periplasmic FRET assay was used to show the *in vivo* interaction dynamics of the d,d-CPases PBP5, PBP6a, and PBP6b. PBP5 and PBP6a were shown to form homodimers, but this could not be directly established for PBP6b. Antibiotic treatment with amdinocillin or aztreonam increased the FRET efficiencies for these interactions, suggesting a different conformation of less active d,d-CPases. Active-site mutants PBP5^S44G^, PBP6a^S66G^, and PBP6b^S63G^ (28, 29) revealed increased FRET efficiencies, confirming this hypothesis. This change in FRET efficiency could be used to monitor d,d-CPase-specific inhibition *in vivo* by new compounds. In general, loss of protein interactions is likely a sign of inhibition of activity (34). To facilitate screening for antibiotics that affect protein interactions, the cuvette-based periplasmic FRET assay was converted into a 96-well plate reader format.

## RESULTS AND DISCUSSION

### Selection and expression of periplasmic fluorescent proteins.

**FIG 1:**
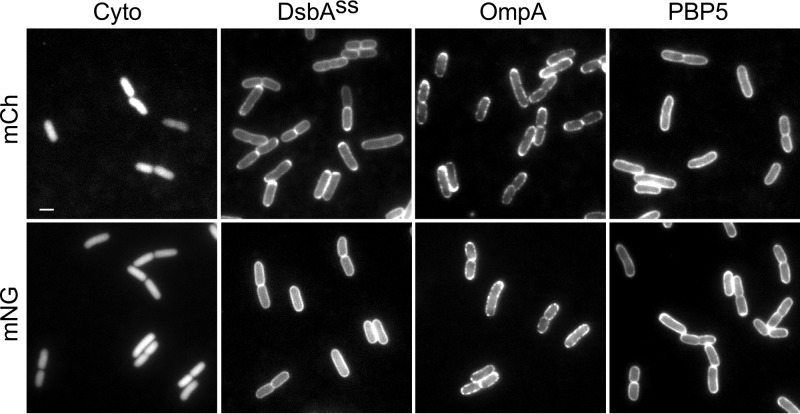
mNG folds and matures in the periplasm. Strain LMC500 was grown in TY at 37°C expressing mCh (top) and mNG (bottom). From left to right, images show expression of cytoplasmic FPs, fusions to the DsbA signal sequence for free-floating periplasmic localization, OmpA-FP for periplasmic OM localization, and FP-PBP5 for periplasmic IM localization. Periplasmic localization of mCh and mNG exerts itself as a halo of fluorescence around the cells. The scale bar equals 2 µm.

The Gram-negative periplasm is an oxidative environment in which most FPs do not fold properly and, thus, do not fluoresce (41, 44, 45). sfGFP and mCherry have been previously reported to fold and mature in the periplasm and could function as a FRET pair. However, their spectral overlap and fluorescent properties, which result in a Förster radius (*R*0) of 5.2 nm, are not optimal (see Text S1 in the supplemental material). Searching for a better donor, we examined the suitability of mNG to function as a periplasmic marker. mNG is a derivative of lancelet fish-based YFP (lanYFP) from *Branchiostoma lanceolatum* (46) and thus unrelated to traditional *Aequorea victoria*-derived FPs, which makes it likely that it has different folding properties than the latter FPs. Compared to sfGFP, mNG has a 40% higher extinction coefficient and a 23% higher quantum yield, leading to higher brightness and a larger *R*0 with mCh (5.5 nm) (Text S1). Fusions of mNG to the DsbA signal sequence (*dsbA*^*ss*^) or to periplasmic proteins clearly localized as fluorescent halos around the cells. mNG replacement of the FP of periplasmic, IM-associated sfGFP-PBP5 or of periplasmic, OM-inserted OmpA-177–mCh fusions (29, 47) resulted in identical fluorescent halos (Fig. 1). These results strongly suggest that mNG is able to fold and mature in the periplasm.

10.1128/mBio.01089-17.1 TEXT S1  Biophysical properties of FPs. Contains Table S1.1 and Fig. S1.1. Download TEXT S1, DOCX file, 0.1 MB. Copyright © 2017 Meiresonne et al. 2017 Meiresonne et al. This content is distributed under the terms of the Creative Commons Attribution 4.0 International license.

Periplasmic proteins are synthesized in the cytoplasm and inserted into or translocated across the cytoplasmic membrane by the SecYEG translocon (48) or the TAT system, which transports folded proteins (49). The TAT system was disregarded as a means to transport our FP fusion proteins since in our experience, penicillin binding proteins especially will interact with their substrate that is synthesized in the cytoplasm and therefore become inaccessible to the Tat system (29). In addition, cytoplasmic FPs were to be avoided, as they also contribute to the fluorescence spectra. Upon Sec-mediated translocation, folding in the periplasm, as well as subsequent processing, is assisted by chaperones (50, 51). However, the expression of exogenous proteins in the periplasm can be problematic and often leads to poorly understood toxicity issues (52, 53).

To study functional interactions with FP fusion proteins, endogenous expression levels should be mimicked to avoid inducing toxicity and artifacts. Overexpression can lead to interactions that occur by random encounters instead of functional interactions (bystander FRET) (54). On the other hand, sufficient expression of the FP fusion is needed to achieve the highest possible fluorescence signal-to-noise ratio. To find the optimal induction conditions, the expression of fusion proteins from a plasmid was induced by various concentrations of isopropyl-β-d-thiogalactopyranoside (IPTG) during growth in a plate reader. Cells grown in rich medium at 37°C that expressed FPs in the cytoplasm grew like wild-type cells that harbored the empty plasmids, i.e., did not express an FP fusion. In contrast, periplasmic expression of mNG via a native fusion protein or the DsbA translocation signal sequence resulted in delayed or impeded growth upon IPTG induction. To a lesser extent, the same effects were observed for cells expressing periplasmic mCh from the same plasmid background (Text S2). Aberrantly growing cultures that did recover showed a delayed but normal growth pattern over time and had lost endpoint fluorescence, suggesting that selection occurs against the periplasmic FP fusions. This indicated that the translocation of mNG fusion proteins to the periplasm was especially toxic to the cells and that counterselection had occurred. Translocation of sfGFP appeared to be equally toxic or worse. Only when the optical density at 600 nm (OD_600_) was kept below 0.3 during growth in rich medium at 37°C and the expression of periplasmic FPs was induced with less than 16 µM IPTG did the cells show no growth defects or effects on fluorescence intensities (Text S2). Cytoplasmic FRET assays are performed with cells grown in glucose minimal medium (Gb1) (71) at 28°C in which the growth rate is slower and for which autofluorescence is less of an issue than for cells grown in rich medium (32). Under these conditions, no toxicity was observed for the periplasmic constructs below 64 µM IPTG induction, indicating that the lower growth rate and temperature largely prevented the previously observed toxicity and allowed good endpoint fluorescence levels (Text S2). Consequently, all periplasmic FRET experiments were performed with cells grown at 28°C in Gb1 flask cultures that were moderately induced with 15 to 20 µM IPTG (Text S2).

10.1128/mBio.01089-17.2 TEXT S2  Toxicity by periplasmic expression. Contains Fig. S2.1 to S2.3. Download TEXT S2, DOCX file, 1 MB. Copyright © 2017 Meiresonne et al. 2017 Meiresonne et al. This content is distributed under the terms of the Creative Commons Attribution 4.0 International license.

### Periplasmic and cytoplasmic mNeonGreen and mCherry spectra match.

**FIG 2:**
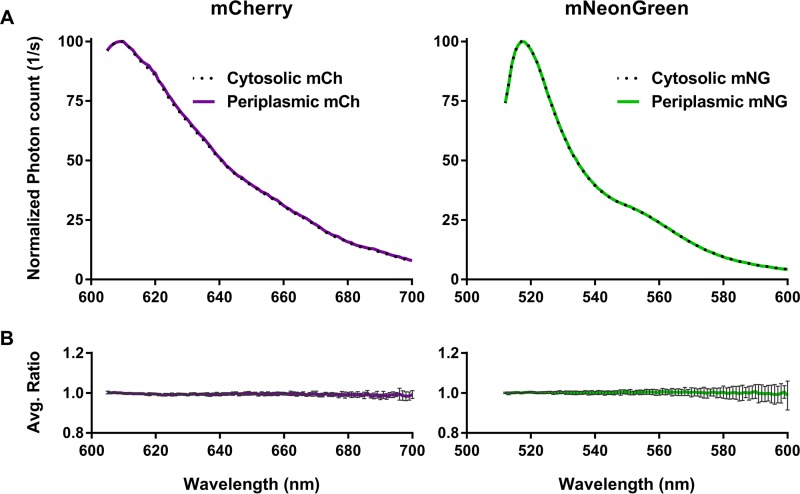
Periplasmic fluorescence emission spectra of mCh (left) and mNG (right) are nearly identical to the spectra of their cytoplasmic counterparts. (A) Normalized emission of mCh (purple) and mNG (green) measured in LMC500 grown for 35 mass doublings in Gb1 at 28°C and induced for two mass doublings. Cytoplasmic (dotted line) and periplasmic (solid line) expression was induced with 50 and 20 µM IPTG, respectively. (B) Average ratio of the normalized cytoplasmic over periplasmic emission spectra from multiple experiments in multiple strains (LMC500, BW25113, MG1655, and CS12-7). For mCh, *n* = 10, and for mNG, *n* = 11; error bars represent the standard deviations.

The periplasmic compartment is an oxidative environment in which the folding of most FPs is impaired (41, 44). In addition, fluorescence excitation and emission spectra can be dependent on the environment in which the FP resides (55). To verify that environmental differences between the cytoplasm and the periplasm did not affect the fluorescence emissions of mNG and mCh, we compared their fluorescence spectra expressed in both compartments of the wild-type strain LMC500. The spectra were measured, and the background spectra of cells containing plasmids that did not express any FP were subtracted before peak normalization. The spectra of FPs expressed in the periplasm and cytoplasm overlapped completely (Fig. 2A), with an average ratio of one, as is expected for identical fluorescence spectra (Fig. 2B). This suggests that the periplasmic and cytoplasmic emission spectra of mNG and mCh are identical and their spectral characteristics (46, 56) can be used to calculate both cytoplasmic and periplasmic FRET efficiencies.

### Validation of the mNeonGreen-mCherry FRET pair.

FRET has been shown to be a useful method to determine functional *in vivo* protein-protein interactions in the *E. coli* cytoplasm (32–36). A minimal FRET experiment requires cells expressing empty plasmids to determine background autofluorescence and cells expressing mNG or mCh, in addition to an accompanying empty plasmid to measure their respective reference spectra. In addition, positive and negative controls were added to each experiment to assess the quality of the measurement and the amount of bystander FRET. We constructed a diffusely localizing cytoplasmic tandem and an OM-attached periplasmic tandem of mNG-mCh as positive controls. A technical negative control for periplasmic FRET is the combination of an IM-bound FP and OM-bound FP that are not supposed to interact and, thus, give no FRET signal. Additional negative controls to determine the amount of bystander FRET consisted of noninteracting protein pairs that are present in the same compartment.

**FIG 3:**
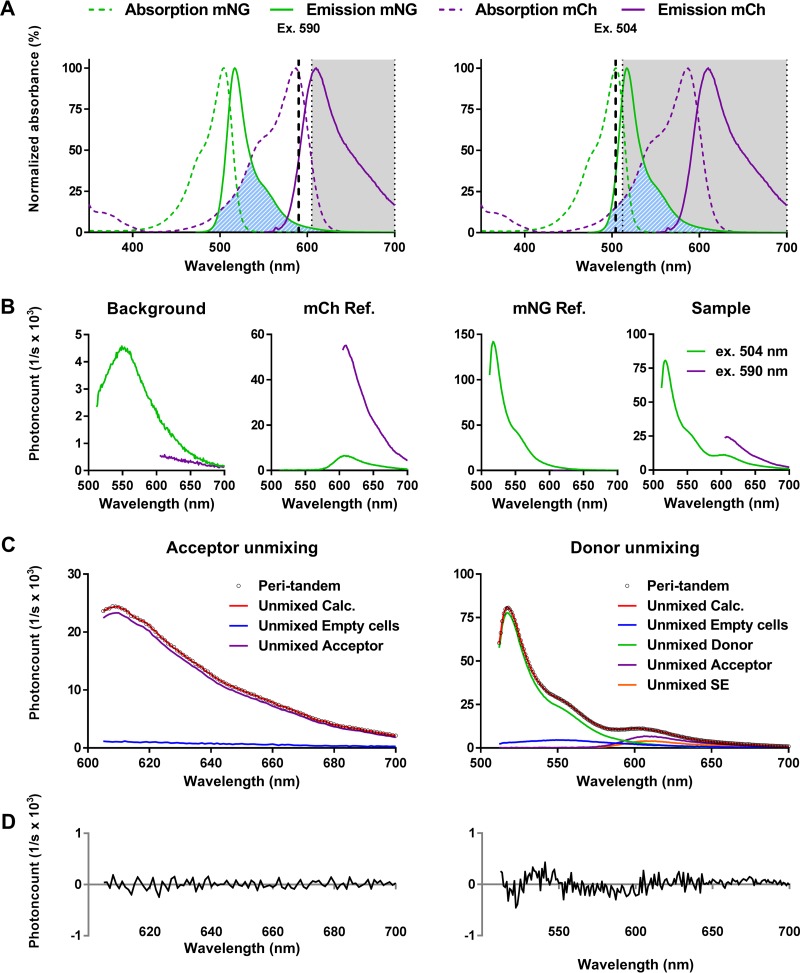
Principle of the periplasmic FRET assay. (A) Excitation and emission spectra of mCh and mNG, indicating the wavelengths used to measure the acceptor channel (left) or the donor channel (right). The hatched blue area represents the spectral overlap between the mNG emission spectrum and the mCh excitation spectrum. The gray shaded area represents the wavelengths for which the emission was measured for the donor or the acceptor. (B) Samples measured to calculate periplasmic FRET. References of background, mCh, and mNG are needed to calculate their contributions to the FRET within a sample. (C) Unmixing of the periplasmic tandem FRET sample, showing the measured spectrum as black dots (Peri-tandem) and the calculated spectrum as a solid red line (Unmixed Calc.). The measured spectrum for the acceptor is composed of the background fluorescence (Unmixed Empty cells) and the amount of mCh (Unmixed Acceptor) present in the sample excited at 590 nm. The measured spectrum for the donor contains the background (Unmixed Empty cells), mNG (Unmixed Donor), and mCh (Unmixed Acceptor) fluorescence and sensitized emission (Unmixed SE), which is the extra fluorescence that the unmixing algorithm cannot attribute to direct excitation of mCh. (D) The low residual difference between the measured and calculated spectra is a measure of the quality of unmixing.

After growth, induction of FP expression, and fixation of the cells, the samples were diluted to an OD_450_ value of 1.00 and fluorescence spectra were measured. mCh was excited at 590 nm, and its emission was measured from 605 to 700 nm (Fig. 3A). To determine the absolute amount of mCh in the sample, the measured spectrum was dissected into its individual components, consisting of background fluorescence and mCh emission, by spectral unmixing using the reference spectra (Fig. 3B and C). Subsequently, the sample was excited at 504 nm, which excites mNG close to its absorption maximum but also directly excites mCh for 15% of its maximum absorbance, and the emission was recorded from 512 to 700 nm (Fig. 3A). Knowing the amount of mCh present in the sample and the expected signal derived from direct mCh excitation, the sample spectrum excited at 504 nm can be unmixed into a background spectrum, an mNG spectrum, and an mCh spectrum using the reference spectra (Fig. 3C and B). Any additional mCh signal can then be attributed to FRET between the mNG donor and the mCh acceptor (Fig. 3C).

Using our mNG-mCh unmixing algorithm, based on mKO-mCh FRET unmixing (32), we calculated the acceptor FRET efficiencies (Ef_*A*_) of the cytoplasmic and periplasmic mNG-mCh tandem fusions using cytoplasmic and periplasmic references. Both mNG-mCh tandems are capable of efficient FRET, as we found Ef_*A*_ values of 17.9% ± 3.0% (mean ± SD) and 15.9% ± 1.6%, respectively (Table 1). The small amplitude of the fit residuals indicates a high quality of measurement (Fig. 3D). Strikingly, the calculated FRET efficiencies were the same independent of whether the cytoplasmic or the periplasmic references were used (Text S3), further supporting the unaltered spectral properties of mNG and mCh in the periplasm.

10.1128/mBio.01089-17.3 TEXT S3  Periplasmic versus cytoplasmic FP references. Contains Fig. S3.1. Download TEXT S3, DOCX file, 0.04 MB. Copyright © 2017 Meiresonne et al. 2017 Meiresonne et al. This content is distributed under the terms of the Creative Commons Attribution 4.0 International license.

**TABLE 1:** FRET efficiencies as calculated from spectral FRET measurements

Parameter	Proteins expressed using plasmid:		Ef_*A*_ (%)^a^	SD	No. of samples tested
	pSAV057	pTHV037			
Positive controls					
Cytoplasmic tandem	Empty vector	Cyto-mNG-mCh	17.9	3.0	10
Periplasmic tandem	OmpA-177SA–mNG–mCh	Empty vector	15.9	1.6	36

Negative controls					
IM/OM	OmpA-177SA–mCh	mNG-PBP5	0.1	0.3	12
	OmpA-177SA–mNG	mCh-PBP5	0.3	0.2	10
OM/OM crowding	TolC-mNG	OmpA-117SA–mCh	0.3	0.1	9
	TolC-mCh	OmpA-117SA–mNG	0.2	0.1	7
IM/IM crowding	FtsN-mCh	mNG-PBP5	0.7	0.1	18
	mNG-PBP5	FtsN-mCh	1.1	0.1	4

Biological interactions					
PBP5-PBP5	mNG-PBP5	mCh-PBP5	2.0	0.5	40
PBP5-PBP5 + amdinocillin	mNG-PBP5	mCh-PBP5	2.7	0.6	8
PBP5-PBP5 + aztreonam	mNG-PBP5	mCh-PBP5	3.7	0.3	6
PBP5^S44G^-PBP5^S44G^	mNG-PBP5^S44G^	mCh-PBP5^S44G^	5.4	0.7	21
PBP6a-PBP6a	mNG-PBP6a	mCh-PBP6a	3.2	0.4	6
PBP6a-PBP6a + amdinocillin	mNG-PBP6a	mCh-PBP6a	3.4	0.7	2
PBP6a^S66G^-PBP6a^S66G^	mNG-PBP6a^S66G^	mCh-PBP6a^S66G^	3.8	0.2	6
PBP6b-PBP6b	mNG-PBP6b	mCh-PBP6b	1.4	0.3	6
PBP6b-PBP6b + aztreonam	mNG-PBP6b	mCh-PBP6b	2.3	0.2	2
PBP6b^S63G^-PBP6b^S63G^	mNG-PBP6b^S63G^	mCh-PBP6b^S63G^	2.0	0.3	6

a For an overview of the unmixing data related to these Ef_*A*_ values, see Text S4 in the supplemental material.

The higher FRET efficiency of the cytoplasmic tandem in comparison to that of the periplasmic tandem suggests that the tandems may not be totally identical. This could be due to a slightly different folding of the periplasmic mNG-mCh tandem, which would result in small differences in the mNG-to-mCh signal ratio. An alternative explanation might be differences in crowding conditions that could lead to a bias in the angle between the donor and acceptor pair, affecting FRET efficiency (54). Technical negative controls with the donor and acceptor FPs attached to the OM and IM or molecular-crowding controls with two unrelated FP fusions localizing to the IM or OM resulted only in low Ef_*A*_ values (Table 1). These results show that our FRET assay with the mNG-mCh FP pair can be used to detect periplasmic protein-protein interactions with Ef_*A*_ values above 1.4% (µ + 3σ) with near certainty. For an overview of the unmixing data, see Text S4.

10.1128/mBio.01089-17.4 TEXT S4  Unmixing of spectrum-based FRET. Contains Fig. S4.1. Download TEXT S4, DOCX file, 1.1 MB. Copyright © 2017 Meiresonne et al. 2017 Meiresonne et al. This content is distributed under the terms of the Creative Commons Attribution 4.0 International license.

### Periplasmic FRET by fluorescence lifetime-imaging microscopy.

To validate our spectrum-based periplasmic FRET assay, we determined FRET by fluorescence lifetime measurements. Fluorescence lifetime-imaging microscopy (FLIM) in combination with time-correlated single-photon counting (TCSPC) (57) was used to measure the fluorescence lifetimes of mNG in the presence and absence of mCh. mNG has a biexponential fluorescence lifetime decay pattern with an average lifetime of 2.7 ns and a dominant lifetime of 3.1 ns for the purified protein (46). Samples of bacterial cells previously used for spectrum-based FRET were spotted on a 1% agarose pad and imaged using the FLIM microscope. The OM-associated periplasmic mNG had an average lifetime of 2.71 ± 0.03 ns composed of a dominant component of 3.01 ± 0.01 and a second component of 1.76 ± 0.12 ns, which is similar to what has been observed in eukaryotic cells (Text S5) (46).

10.1128/mBio.01089-17.5 TEXT S5  Fluorescence lifetime-based FRET. Contains Fig. S5.1 and Table S5.1. Download TEXT S5, DOCX file, 0.9 MB. Copyright © 2017 Meiresonne et al. 2017 Meiresonne et al. This content is distributed under the terms of the Creative Commons Attribution 4.0 International license.

FLIM measures the average lifetime of a population of both radiative and nonradiative decaying donor FPs. The average lifetime of a donor FP decreases when FRET occurs because it loses more energy to the acceptor by the energy transfer, and therefore, the chance to detect longer-lived excited FPs that fluoresce decreases. In the absence of FRET, there is no additional relaxation pathway for the excited FP and the average lifetime of the donor FP will be the same as if no acceptor was present.

When mCh is photobleached by scanning with a high-intensity 561-nm laser, the bleached acceptor is unable to accept energy from the mNG donor molecule. Therefore, upon total bleaching of the mCh acceptor molecule in the upper half of a field of view, the donor fluorescence lifetime in FRET samples should be restored to the donor-only lifetime. This makes FLIM a good technique to measure or confirm periplasmic FRET, as it requires only one sample slide that will have its own internal control. For an overview, see Text S5. Periplasmic mNG lifetimes from the unbleached region in the absence of mCh were not significantly affected by the acceptor photobleaching protocol, with average amplitude-based lifetime (τ_amp_) values of 2.73 ± 0.02 ns versus 2.74 ± 0.03 ns, respectively. The periplasmic tandem showed an increase of the donor fluorescence lifetime upon bleaching, from τ_amp_ values of 2.22 ± 0.04 ns to 2.64 ± 0.06 ns, indicating that the fluorescent proteins in the cells within the unbleached region were transferring energy by fluorescence resonance. This effect is significant compared to the fluorescence lifetimes of the technical-negative-control group using periplasmic but physically separated mNG and mCh molecules that were unaffected by photobleaching, with τ_amp_ values of 2.62 ± 0.03 ns and 2.63 ± 0.03 ns for unbleached and bleached regions, respectively. Calculating the FRET efficiencies of the periplasmic tandem and the negative control using the formula FRET efficiency = 1 − (τ_amp_ unbleached/τ_amp_ bleached) (58) results in FRET percentages of 15.8% ± 1.3% and 0.6% ± 1.0%, respectively (Text S5). These efficiencies are similar to the results observed by spectrum-based FRET, validating both techniques for measuring periplasmic protein interactions.

### Scalability of periplasmic FRET.

**FIG 4:**
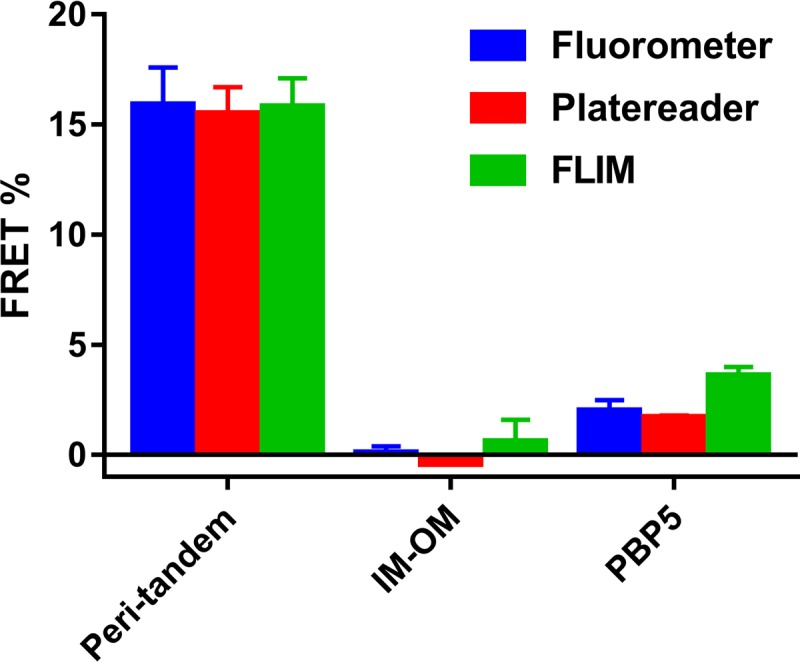
Comparison of the FRET efficiencies measured by different methods. Fluorometer and plate reader FRET efficiencies are measured by spectrum-based techniques, and FLIM-FRET efficiencies by fluorescence lifetime. Associated FRET efficiencies are shown in Table 1; see also Text S5 and S6.

To work toward an assay suitable for higher-throughput screening purposes, the periplasmic FRET assay was scaled up to a 96-well plate assay. Samples that were used in a regular FRET experiment were dispensed into a 96-well plate, and fluorescence spectra were measured using a fluorescence plate reader. Using a modified unmixing algorithm, the Ef_*A*_ values from the fixed samples in the plate reader could be calculated much faster than those measured in single cuvettes and gave similar results. Subsequently, the periplasmic FRET assay procedure was performed completely *in vivo* by growing and inducing cultures in the plate reader and measuring fluorescence spectra directly from the living cells (Text S6). Generally, the end OD_450_ values of the replicates were within 10% of each other for each group and the average spectrum intensities did not differ more than ∼15% from each other. For an overview of the plate reader FRET unmixings, see Text S7. The resulting Ef_*A*_ values were highly similar to those of the fixed single-sample FRET and FLIM-FRET (Fig. 4; Text S6). This shows that the periplasmic FRET assay can be used with living cells in a medium-throughput assay.

10.1128/mBio.01089-17.6 TEXT S6  Plate reader FRET. Contains Fig. S6.1 to S6.2 and Table S6.1. Download TEXT S6, DOCX file, 1.2 MB. Copyright © 2017 Meiresonne et al. 2017 Meiresonne et al. This content is distributed under the terms of the Creative Commons Attribution 4.0 International license.

10.1128/mBio.01089-17.7 TEXT S7  Supplemental materials and methods. Download TEXT S7, DOCX file, 0.03 MB. Copyright © 2017 Meiresonne et al. 2017 Meiresonne et al. This content is distributed under the terms of the Creative Commons Attribution 4.0 International license.

### *In vivo* oligomerization of d,d-CPases.

With the periplasmic FRET assay established and validated, it can be employed to identify and characterize biological interactions *in vivo*, for which the class C PBPs PBP5, PBP6a, and PBP6b are ideal candidates. These highly similar d,d-CPases are translated as preproteins with N-terminal signal sequences that are cleaved off after translocation into the periplasm through the Sec translocon (28). Periplasmic d,d-CPases associate with the outer leaflet of the IM via their C-terminal amphipathic helices (20). PBP5 is the most prevalent d,d-CPase and has already been suggested to form homodimers based on *in vivo* cross-linking experiments (59). The absence of PBP5 in a Δ*dacA* strain can be complemented by FP-PBP5 expressed from a plasmid that localizes, like the endogenous protein, primarily at midcell during cell division and to the lateral wall in general (29). Samples of cells expressing mNG-PBP5 and mCh-PBP5 gave significant Ef_*A*_ values of 2.0% ± 0.5% (Table 1), proving *in vivo* PBP5 interactions using the periplasmic FRET assay. The PBP6a self-interaction assayed with the mNG-PBP6a and mCh-PBP6a pair resulted in Ef_*A*_ values of 3.2% ± 0.4%. The PBP6b self-interaction measured with the mNG-PBP6b and mCh-PBP6b pair resulted in Ef_*A*_ values of 1.4% ± 0.2%, which are at the cutoff value for bystander FRET (Table 1).

### Inhibition of class B PBPs causes a rearrangement of d,d-CPase dimer structures.

Inhibition of PBP2 by amdinocillin (60) reduces the activity of its cognate class A protein PBP1a (61), and inhibition of PBP3 by aztreonam (60) might also affect its partner protein, PBP1b (62). Consequently, these antibiotics will likely reduce the availability of pentapeptide substrate for d,d-CPases, even though the proteins themselves are not directly inhibited (60). To investigate whether the reduced activity of d,d-CPases affects their interaction, cells expressing PBP5, PBP6a, and PBP6b FRET constructs were grown in the presence of amdinocillin or aztreonam. Compared to the Ef_*A*_ values observed for the active PBP5 proteins, we found increased Ef_*A*_ values of 2.7% ± 0.6% and 3.7% ± 0.3% for amdinocillin- and aztreonam-treated cells, respectively. Amdinocillin treatment of wild-type PBP6a resulted in a relatively small increase, with Ef_*A*_ values of 3.4% ± 0.7%, and aztreonam treatment of active PBP6b increased the Ef_*A*_ values to 2.3% ± 0.2%, which is above the crowding cutoff limit (Table 1). The observed changes in FRET efficiency for d,d-CPases under d,d-TPase-inhibited conditions suggest a conformational change of the less active d,d-CPase dimers that orients the chromophores in a more favorable position for FRET.

### Inactive d,d-CPases have a different dimer structure.

To confirm that inactive d,d-CPases have an alternative conformation, the FRET experiments were repeated with the active-site mutants PBP5^S44G^, PBP6a^S66G^, and PBP6b^S63G^ (28, 29, 63). The mutation changing serine to glycine removes the reactive hydroxyl group that would be deprotonated and nucleophilically attacks the carbonyl carbon of the d-Ala-d-Ala substrate, rendering the enzymes inactive. FRET experiments with plasmids expressing the inactive PBP5^S44G^ mutant in the Δ*dacA* strain yielded an Ef_*A*_ value of 5.4% ± 0.7%, which is a large increase compared to the wild-type PBP5 Ef_*A*_ value of 2.0%. This is similar to the increase of the Ef_*A*_ value for the PBP5 interaction observed for aztreonam-treated cells, indicating that the inactive form indeed corresponded to the situation in which the class B PBP3 was inhibited (Table 1). The same effect was observed for the PBP6b^S63G^ mutant, which gave an Ef_*A*_ value of 2.0% ± 0.3%, an increase similar to that observed for the wild-type PBP6b cells treated with aztreonam. The differences in Ef_*A*_ values between active and inactive PBP6b were smaller than for PBP5, which is expected given that PBP6b is reported to be more active at pH 5.0 (26). The FRET efficiency for the PBP6a^S66G^ active-site mutant was only moderately increased relative to that of the wild-type PBP6a protein, with Ef_*A*_ values of 3.8% ± 0.2% (see below). These results suggest that inactivation of d,d-CPases changes the conformation of their dimeric state. Inhibition of class B PBPs seemed to decrease the activity of d,d-CPases, presumably by reducing the number of available pentapeptides or nascent PG. Consequently, the activities of the class A and class B PBPs are likely coupled *in vivo*, confirming the *in vitro* evidence (61).

### d,d-CPase active-site mutants show different localization patterns.

**FIG 5:**
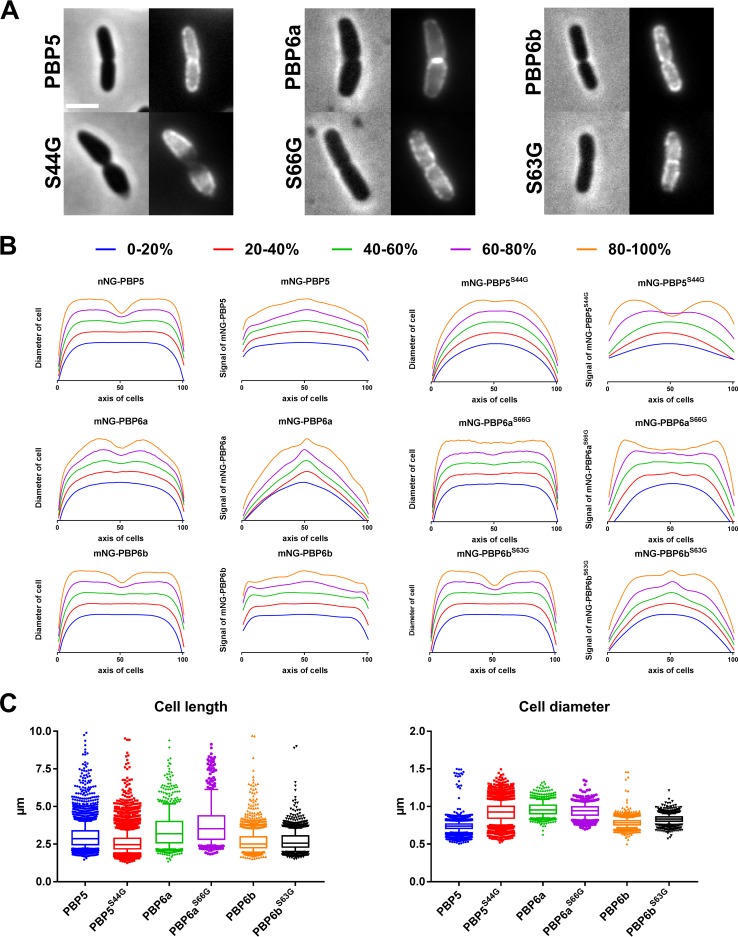
d,d-CPases and their active-site mutants show different localization patterns when grown under poor medium conditions. (A) (Top) Active PBP5 and PBP6b localize more weakly than PBP6a at midcell in the Δ*dacA* strain. (Bottom) Their inactive mutants show different localization patterns. The scale bar equals 2 µm. (B) Quantification of the cellular morphology of cells separated into 20% age (progression of cell cycle) groups to qualitatively compare cell shape and fluorescence signals as a function of the cell cycle (70). This reveals a regular shape for the Δ*dacA* cells expressing PBP5 or PBP6b despite weak midcell localization. Interestingly, the cells expressing midcell-localizing PBP6a show a swollen morphology. PBP5^S44G^ avoids the midcell position and the poles, while PBP6a^S66G^ avoids the midcell and seems to accumulate in the first and last quarter of the cells. The cells expressing PBP5^S44G^ have a swollen morphology, while the cells expressing PBP6a^S66G^ seem to have a minor invagination defect. The PBP6b^S63G^ signal is enhanced at the midcell position compared to that of its active counterpart, but the cells show no differences in morphology. The horizontal axes of the graphs represent the normalized lengths of the cells, diameters of cells reflect peak normalized diameters, and signals of FP-fusions represent peak normalized fluorescence intensities. The numbers of cells analyzed were as follows: mNG-PBP5, *n* = 3,908; mNG-PBP5^S44G^, *n* = 4,401; mNG-PBP6a, *n* = 784; mNG-PBP6a^S66G^, *n* = 487; mNG-PBP6b, *n* = 1,754; and mNG-PBP6b^S63G^, *n* = 1,585. (C) Boxplots of the measured cell lengths and diameters for each culture show a decrease in cell length for cells expressing PBP5^S44G^, PBP6b, or PBP6b^S63G^; these cells have an increased diameter. The cells expressing PBP6a or PBP6a^S66G^ were longer and thicker. The whiskers represent the 10th and 90th percentiles.

*In vivo*, d,d-CPase activity is abolished in the PBP5^S44G^, PBP6a^S66G^, and PBP6b^S63G^ mutants (28, 29, 63). Conceivably, all mutants have a lower affinity for their substrate, as inability to bind substrate was reported for PBP5^S44G^ *in vitro* (63). If none of the mutants exert d,d-CPase activity or substrate binding, perhaps other factors play a role in localization. When mCh-PBP5, mCh-PBP6a, and mCh-PBP6b or their inactive variants were expressed in Δ*dacA* cells in minimal medium, the results were different from the results in cells grown in rich medium (28, 29). In minimal-medium-grown cells, PBP5 localized laterally and at the midcell, whereas PBP5^S44G^ only localized laterally, avoiding the midcell (Fig. 5). PBP6a localization was lateral and intense at the midcell, but this was not observed for PBP6a^S66G^, which mostly avoided the midcell (Fig. 5B). PBP6b localized poorly at the midcell and mainly at the lateral sides, but PBP6b^S63G^ still showed midcell fluorescence. Interestingly, the phenotypes of wild-type PBP5 and the inactive PBP6a^S66G^ mutant seemed relatively normal, while in the reverse situation, i.e., inactive PBP5^S44G^ and wild-type PBP6a, the phenotypes were affected. This suggests that a balanced activity of PBP5 and PBP6a is likely a requirement for morphology maintenance.

Inactive mNG-PBP6a^S66G^ and mNG-PBP6b^S63G^ displayed an interesting localization pattern that was not observed for mNG-PBP5^S44G^. In some cells, they were present as a thin band exactly at the midcell in an area where they were otherwise absent (Fig. 5A). Whether this was due to high levels of substrate availability or because they were aided by other factors remains to be elucidated. Comparing the phenotypes of the PBP5^S44G^, PBP6a^S66G^, and PBP6b^S63G^ mutants, it was mainly the PBP5^S44G^ mutant that showed a bulging phenotype around the plane of cell division. This indicates that the PG layer was not able to fully withstand the internal turgor pressure and that its organization was in one way or another impaired. Further quantification of cell morphology by cell length and diameter revealed that all cells without active PBP5 had greater diameters (Fig. 5C). Compared to the results for cells expressing wild-type PBP5, the expression of PBP5^S44G^ resulted in reduced cell lengths, but PBP6a and PBP6a^S66G^ expression resulted in longer cells. For both cell length and cell diameter, the expression of PBP6b partially restored the wild-type PBP5 situation, possibly because the optimal conditions for PBP6b were not met (26). With these results, we revisit and add to previous work by the Kevin Young laboratory (28) and underscore the need to discriminate between the presence of inactive d,d-CPases and the absence of activity in d,d-CPase deletion strains.

### Inactive PBP5^S44G^ affects morphology.

**FIG 6:**
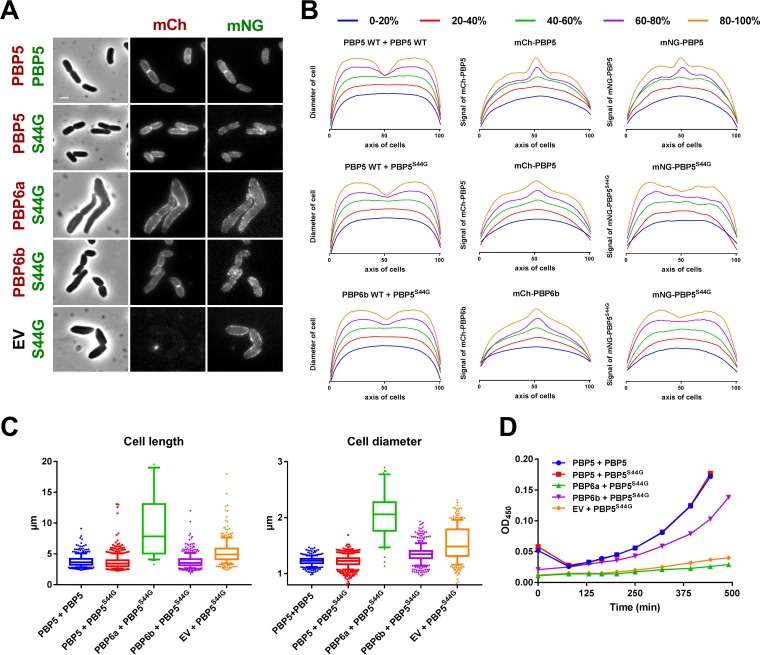
The PBP5^S44G^ overexpression phenotype is partially prevented by coexpressing PBP6b but not by PBP6a. (A) Δ*dacA* cells grown in rich medium expressing PBP5^S44G^ show the swollen phenotype and absence of fluorescence at the poles and at midcell, which is not observed when expressing only FP-PBP5. The coexpression of FP-PBP5 with the mutant prevents the phenotype, but the (de)localization of fluorescence remains the same. The same effect is observed with PBP6b but not with PBP6a, which results in an exacerbated phenotype. On the left, the expressed fusion proteins are represented and colored red for mCh fusions and green for mNG fusions. S44G, PBP5^S44G^; EV, empty vector not expressing an FP fusion. The scale bar represents 2 µm. (B) The normalized diameter profiles of cells where the PBP5^S44G^ phenotype was complemented with PBP5 or PBP6b show a similar invagination pattern as for PBP5-only cells. The normalized fluorescence profiles of PBP5 fusions display midcell localization for dividing cells irrespective of coexpression with PBP5^S44G^, as does PBP6b. The fluorescence profiles of PBP5^S44G^ do not localize at midcell regardless of active d,d-CPase coexpression. The horizontal axes of the graphs represent the normalized lengths of the cells, diameters of cells reflect peak normalized diameters, and signals of FP-fusions represent peak normalized fluorescent intensities. The numbers of cells analyzed for each group are as follows: PBP5+PBP5 = 323, PBP5^S44G^+PBP5 = 465, and PBP5^S44G^+PBP6b = 398. (C) Boxplots of the cell lengths and diameters show that expression of inactive PBP5^S44G^ results in an elongated, thicker phenotype and that coexpression with wild-type PBP5 reverses this effect. Coexpression with wild-type PBP6a worsens the phenotype, and PBP6b partially restores a wild-type phenotype. The numbers of cells analyzed are as shown above, with the addition of PBP6a+PBP5^S44G^ (*n* = 44) and EV+PBP5^S44G^ (*n* = 295). (D) The Δ*dacA* cells grown in poor medium also show growth rate complementation of the PBP5^S44G^ phenotype by wild-type PBP5 and, to a lesser extent, by PBP6b.

The absence of d,d-CPases is not the same as their inactive presence. While a Δ*dacA* morphology is like the WT morphology and the expression of FP-PBP5 in the Δ*dacA* strain also provides a WT phenotype, the expression of FP-PBP5^S44G^ eventually results in cells bulging at the midcell (Fig. 6A). When wild-type FP-PBP5 and FP-PBP5^S44G^ are coexpressed, the active fusion protein independently localizes at the midcell and at the lateral wall, whereas the mutant localizes only at the lateral wall and the cells have WT morphology (Fig. 6B). Cell division is accompanied by active PG synthesis at the midcell, and PBP5 localization is dependent on substrate availability (29). The lack of substrate affinity of FP-PBP5^S44G^ allows the active FP-PBP5 to prevent the bulging phenotype caused by expressing FP-PBP5^S44G^. Interestingly, complementation was not achieved by chromosomally encoded PBP6a or PBP6b, possibly because their expression or activity conditions were not met (26). The Δ*dacA* strain was therefore transformed with plasmids encoding mCh-PBP6a or mCh-PBP6b in combination with mNG-PBP5^S44G^ and expression was induced in rich medium, after which the cells were imaged. The FP-PBP5^S44G^ shape defects could be partially mitigated by coexpression of FP-PBP6b, but FP-PBP6a exacerbated this morphology. The FP-PBP6b that was ameliorating the FP-PBP5^S44G^ phenotype localized at the midcell (Fig. 6A and B). Apparently, PBP6b is localizing strongly when PG synthesis is affected at the midcell and is able to provide some of the functionality of PBP5. Further quantification of the cell lengths and cell diameters confirms the complementation of the PBP5^S44G^ phenotype by PBP5 and partially by PBP6b and its exacerbation in combination with PBP6a (Fig. 6C). In the absence of (partially) complementing expression of another d,d-CPase, cells expressing PBP5^S44G^ had a reduced growth rate (Fig. 6D).

Treatment of cells with aztreonam increases the FRET efficiency for the PBP5 self-interaction, as does mutating its active site. These data suggest that, indeed, inactive PBP5 cannot bind the pentapeptide substrate at the midcell and resides laterally in its inactive conformation the same way wild-type PBP5 does when PBP3 inhibition limits the availability of its substrate. Curiously, Δ*dacA* cells expressing PBP5 that are treated with aztreonam are filamentous but can maintain their diameter, whereas cells expressing PBP5^S44G^ bulge. The PBP5^S44G^ phenotype is caused by the presence of the mutant and not by its inactivity, as cells without PBP5 activity due to its absence (Δ*dacA*) show only minor morphology defects. These results suggest that inactive PBP5 unbalances processes involved in PG cross-linking. The cells become swollen, suggesting either that PG is less cross-linked or that the accuracy of cell diameter regulation is lost. Since the inactive PBP5 is not present at the midcell, the observed morphological changes could be caused either by titration of divisomal proteins from the midcell or interference with the elongation machinery.

### Model for d,d-CPase conformational changes.

**FIG 7:**
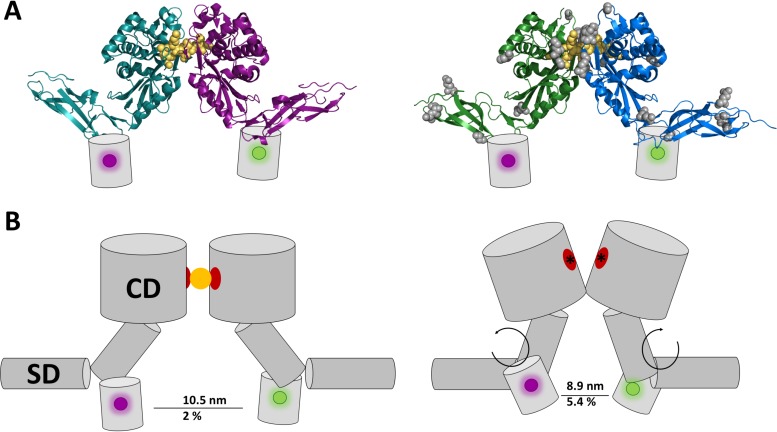
Model of the preacylation dimer and the nonactive dimer of d,d-CPases. (A) (Left) Structure of the preacylation complex of PBP6a (PDB ID 3ITB) with its substrate, shown in yellow (66). (Right) Model of PBP5 (PDB ID 5J8X) (18) aligned on the PBP6a structure, with the previously found cross-linked residues indicated by gray spheres (59) and the FPs by gray barrels marked with purple for mCh and green for mNG. (B) (Left) Model of the active PBP5 dimer. (Right) Model of the inactive dimer. The catalytic domain (CD) contains the active site, shown in red, and is connected to the donor or acceptor FP through a flexible stem domain (SD). Inactivity of the enzyme is indicated by asterisks, and substrate is shown as a yellow circle. The increased FRET efficiency for the inactive PBP5 dimer means the distance between the chromophores has shortened to ∼8.9 nm, which could be achieved by a reorientation of the flexible region (59) between the stem and catalytic domains to produce an open conformation without substrate (ready to receive substrate). This open conformation could result in the fused donor and acceptor FPs being closer together or oriented more optimally to allow more energy transfer.

Many crystal structures of PBP5 are available (4, 18, 59, 64, 65), but none of them convincingly forms a dimer in the crystal lattice. These crystals were obtained by cytoplasmic overexpression of the proteins lacking their C-terminal α-helix. Removal of the IM-associating α-helix from PBP5, PBP6a, or PBP6b results in its delocalization from the septum (28, 29) and possibly prevents substrate binding. However, *in vivo* cross-linking of PBP5 suggests that PBP5 is able to dimerize (59). The cross-linkable residues were found distributed over the entire surface of the protein, suggesting that more than one conformation might be available to form a crosslinked dimer. In the dimer model compiled from two soluble proteins without substrate suggested by Skoog et al. (59), the N termini where the FPs are fused are close together, possibly explaining the higher FRET efficiencies of inactive d,d-CPases (Fig. 7).

The only crystal structure of a preacylation complex with the native substrate of a d,d-CPase is that of PBP6a (66). In this structure, the substrate *N*-acetylmuramic acid-(L-Ala-d-*iso*Glu-l-Lys-d-Ala-d-Ala) is shared by the active sites of two PBP6a molecules (Fig. 7A). Analysis of this structure revealed binding of the substrate but not the closed conformation needed for hydrolysis (17). Indeed, kinetic studies found that PBP6a does not hydrolyze the substrate used for the crystal structure (27). It cannot be excluded that PBP6a is enzymatically inactive in the cell and that this serves a purpose. This may explain the higher Ef_*A*_ values found for the PBP6a self-interaction compared to the Ef_*A*_ values for the active PBP5 and, also, the relatively smaller increase upon inactivation by introducing the PBP6a S66G mutation compared to the increase for the PBP5^S44G^ mutant. PBP5 and PBP6a have 59% identical and 76% similar amino acids and are consequently structurally very similar. And yet, their abilities to bind and catalyze the many substrate analogues of the pentapeptide stem are different, and consequently, they probably have different functions while competing for the same substrates. For instance, while PBP5 removes the last d-Ala, PBP6a might be there to protect the last d-Ala from removal, as was recently suggested (26). The balance between PBP5 and PBP6a might affect the type or rate of cross-linking of the PG layer.

### In conclusion.

The periplasm of Gram-negative bacteria contains the PG layer that allows cells to withstand the internal turgor pressure. Inhibition of PG-modifying proteins has long been an attractive target for antibiotics like the β-lactams. Resistance against these antibiotics is increasing, and new intrinsic mechanisms of resistance show the urgency for novel rationally developed antibiotics. Functionality of the otherwise nonessential PG-modifying protein PBP5 was found to be required for β-lactam resistance through alternative cross-linking of PG by proteins that are not targets of most β-lactams. The d,d-CPases PBP5, PBP6a, and PBP6b are thus attractive targets for inhibition to prevent escape from β-lactam treatment.

Determining the interactions of these proteins *in vivo* has been a challenge, as no useful methods were available. Here, we present a FRET-based assay to quantitatively and qualitatively measure periplasmic protein-protein interactions *in vivo*. We have shown the relatively novel fluorescent protein mNG to fold and mature in the periplasm. mNG is a compatible FRET donor for use with mCh, with a calculated Förster radius of 5.5 nm. Neither of these periplasmic FPs exhibits altered fluorescence properties compared to those of its cytoplasmic version. We established and validated our periplasmic FRET assay with positive and negative controls by spectral and fluorescence lifetime-based techniques and increased its throughput to 96-well plates.

Employing the periplasmic FRET assay, we showed self-interaction of PBP5, PBP6a, and PBP6b. Our data suggest that active and nonactive d,d-CPases form structurally different dimers. Inhibition of class B PBPs likely reduced the substrate available for d,d-CPases, and this caused increases in their self-interaction FRET efficiencies. Inactivating the d,d-CPases by mutating their active-site serine confirmed this hypothesis, because the inactive mutants also showed increased self-interaction FRET efficiencies. The inactive d,d-CPase mutants behaved phenotypically differently from their wild-type versions. Cells expressing inactive PBP5^S44G^ bulged at the midcell. The bulging phenotype could be prevented by the expression of PBP5 or PBP6b but not PBP6a, suggesting a difference in function. PBP6a may protect the fifth d-Ala from being hydrolyzed during PG cross-linking, as it can bind substrates mimicking the pentapeptide stem but is less capable of hydrolyzing them. By competing for the same substrate, PBP5 and PBP6a may assist in balancing PG synthesis by the class A and B PBPs. Indeed, PBP6a is the only d,d-CPase that is upregulated when the growth rate is reduced under minimal medium conditions, while the others are downregulated (25).

Here, we describe the activity-related conformational changes of d,d-CPases and provide an assay to determine periplasmic protein-protein interactions *in vivo*. As much as 30% of the genome of *Escherichia coli* encodes proteins that are predicted to localize to the cell envelope, and many essential processes, such as nutrient import, chemotaxis, cell shape maintenance, and cell division, function fully or in part in the periplasm (67). The assay presented herein facilitates mode-of-action studies of inhibitors that affect the interaction of proteins that participate in these essential activities in the Gram-negative cell envelope.

## MATERIALS AND METHODS

### Bacterial strains, culture conditions, plasmid construction, toxicity studies, and imaging.

The bacterial strains used in this study are listed in Text S7 in the supplemental material and were grown as described in nutrient-rich TY or nutrient-poor Gb1 medium (Text S7). All plasmids used in this study are variations of the pTHV037 or pSAV057 inducible expression vectors (32, 68) and are listed in Text S8. For a detailed description of their construction, see Text S7 and S8. The toxicity of expressing periplasmic fusion proteins was determined by inducer titration experiments and microscopic imaging of cells expressing cytoplasmic or periplasmic FPs, which were performed as described in Text S7.

10.1128/mBio.01089-17.8 TEXT S8  Strains, plasmids, and cloning strategies. Contains Tables S8.1 to S8.4. Download TEXT S8, DOCX file, 0.1 MB. Copyright © 2017 Meiresonne et al. 2017 Meiresonne et al. This content is distributed under the terms of the Creative Commons Attribution 4.0 International license.

### Spectrum-based FRET.

References, controls, and FRET sample cultures were inoculated into 5 ml TY and grown overnight at 37°C. Subsequently, the cultures were diluted 1:1,000 in Gb1 and grown for approximately 35 mass doublings at 28°C while keeping the OD_450_ values below 0.2. Expression of the fusion proteins was induced with 15 µM IPTG for at least 2 mass-doubling times while keeping the OD_450_ below 0.2, before fixation with a final concentration of 2.8% formaldehyde and 0.04% glutaraldehyde for 15 min in the shaking water bath, after which the cells were harvested. PBP2 was inhibited by 2 µg/ml amdinocillin and PBP3 was inhibited with 1 µg/ml aztreonam simultaneously with the induction of the FRET couple by IPTG for 2 mass doublings where indicated. The cytoplasmic and periplasmic mNG- or mCh-only references were induced by 50 and 20 µM IPTG, respectively. The samples were washed 3 times with 1 ml phosphate-buffered saline (PBS) (69). Before measuring fluorescence spectra, the samples were washed once more with 1 ml PBS and diluted to OD_450_ values of 1.00 ± 0.05. The samples were loaded into a 10- by 4-mm quartz cuvette with a micro-magnetic stirrer (Hellma Analytics, Müllheim, Germany) and placed in a spectrofluorimeter (Photon Technology International, NJ). Acceptor and donor emission spectra were measured through 6-nm slit widths with 1-s integration time per scanned nm and 3 times averaging. For the acceptor (mCh) channel, samples were excited by the monochromator set at 590 nm through a 587-nm ± 11-nm single band-pass (BP) filter (BrightLine; Semrock, Rochester, NY) and emission wavelengths from 605 to 700 nm at 1 nm increments were measured through a 600-nm long-pass (LP) filter (Chroma Technology Corp., Bellows Falls, VT). This spectrum was used to determine the amount of mCh in the sample. For the donor (mNG) channel, samples were excited by the monochromator set at 504 nm through a 500-nm ± 10-nm BP filter (Semrock) and emission wavelengths from 515 to 700 nm at 1-nm increments were measured through a 510-nm LP filter (Chroma). A blank measurement of PBS only was subtracted from all samples, and the empty-cell reference was subtracted from the donor and acceptor spectra to make the FP references as clean and close as possible to their fluorescence-only spectra. Knowing the amount of mCh present in the sample and the shapes of the mCh and mNG spectra and background fluorescence spectrum in the cells, the sample spectra could then be unmixed into their separate components: background fluorescence, mNG, mCh, and sensitized emission (FRET). The FRET efficiencies were calculated with published algorithms (32), using the extinction coefficient and quantum yield for mNG (Text S1) as published previously (46).

### Plate reader spectrum-based FRET.

All cultures were grown to steady state in Gb1 in flasks and then diluted to OD_450_ values of 0.005 in black glass-bottom 96-well plates in a total volume of 200 µl. The cultures then continued to grow in the multimode plate reader at 28°C, set to shake at medium speed. As growth reached OD_450_ values of ∼0.1, the cultures were diluted 1:2 with fresh prewarmed Gb1 medium containing IPTG to an end concentration of 20 µM and growth was continued. Before the cultures reached stationary growth (generally an OD_450_ of 1.0), the fluorescence spectra of the acceptor and donor channels were measured. The plate reader spectral measurements used monochromator-based excitation and emission settings with minimal slit widths of 9 nm using 10 times averaging detected from the bottom side of the plate. For the acceptor mCh channel, samples were excited at 590 nm and emission wavelengths from 610 to 700 nm at 1-nm increments were measured. For the mNG donor channel, samples were excited at 495 nm and emission spectra were collected from 517 to 700 nm at 1-nm increments. Spectra of good-quality wells were pooled and averaged and then used to calculate FRET efficiencies using the spectral unmixing spreadsheet as described in “Spectrum-based FRET” above, with donor parameters for 495 instead of 504 nm.

### Fluorescence lifetime-based FRET.

Fluorescence lifetime images were acquired using an Olympus FV1000 (Olympus, Tokyo, Japan) confocal microscope equipped with a Picoharp TCSPC module (Picoquant, Berlin, Germany). Samples were grown as described in “Spectrum-based FRET” above. Slides prepared as described in Text S7 were mounted on the table, and a field of view of 256 by 256 pixels was illuminated with a pulsed 485-nm Picoquant diode laser (20 MHz, 0.4 kW.cm^−2^) using an Olympus UPLS Apo 60× water numeric aperture 1.2 objective lens. The fluorescence signal was detected in semiconfocal mode with the pinhole diameter set at 200 µm. The fluorescence that passed a 405/480/560/635 dichroic mirror was filtered by a 505- to 540-nm emission filter and detected by using avalanche photodiodes (MPD, Bolzano, Italy).

The presence of acceptor mCh was visualized by illuminating the sample with a 561-nm laser and detecting the emission through a 570-nm LP filter (Olympus). Acceptor photobleaching was achieved by scanning a region of interest with the 561-nm laser for 1 to 2 min at 150 kW.cm^−2^.

In order to obtain a reliable fluorescence lifetime, the measurement times of the mNG donor channel were set such that at least 10^5^ photons were collected. The full fluorescence decay curves were fitted (SymPhoTime 64; Picoquant) using a biexponential decay model that included an instrumental response function generated from the same data set. The fitted results were accepted or discarded on the basis of visual inspection of the fit, the fit residuals, and the minimal chi square.
