# Erratum for Meiresonne et al., “Activity-Related Conformational Changes in d,d-Carboxypeptidases Revealed by *In Vivo* Periplasmic Förster Resonance Energy Transfer Assay in Escherichia coli”

**DOI:** 10.1128/mbio.03577-21

**Published:** 2022-01-18

**Authors:** Nils Y. Meiresonne, René van der Ploeg, Mark A. Hink, Tanneke den Blaauwen

**Affiliations:** a Bacterial Cell Biology and Physiology Swammerdam Institute for Life Sciences, University of Amsterdam, Amsterdam, The Netherlands; b Molecular Cytology and Van Leeuwenhoek Centre for Advanced Microscopy, Swammerdam Institute for Life Sciences, University of Amsterdam, Amsterdam, The Netherlands

## ERRATUM

Volume 8, issue 5, e01089-17, 2017, https://doi.org/10.1128/mBio.01089-17. Text S7 and Text S8 in the supplemental material erroneously stated that the mNeongreen gene used was codon optimized for Escherichia coli. Instead, it was only changed to alter restriction sites. The files have been corrected.

