# Supplementary material for: Activity-Related Conformational Changes in d,d-Carboxypeptidases Revealed by In Vivo Periplasmic Förster Resonance Energy Transfer Assay in Escherichia coli
Source: mBio. 2017 Sep 12;8(5):e01089-17. doi: 10.1128/mBio.01089-17 (PMC5596342; doi:10.1128/mBio.01089-17)
Supplement: TEXT S1 [file mbo004173468s1.docx]

## SI 1 – Physical properties for FRET with mCh, mNG and sfGFP

Contents

Table S1.1 - Spectral properties of sfGFP, mNG and mCh

Fig. S1.1 - Spectral overlaps and Förster radii of mNG and sfGFP with mCh

References

Table S1.1 Spectral properties of sfGFP, mNG and mCh

| FP (reference) | **sfGFP** (1) | **mNeonGreen** (2) | **mCherry** (3) |
| --- | --- | --- | --- |
| Excitation max (nm) | 485 | 506 | 587 |
| Emission max (nm) | 510 | 517 | 610 |
| Extinction coefficient (M^-1^ cm^-1^) | 83300 | 116000 | 72000 |
| Quantum yield | 0.65 | 0.8 | 0.22 |
| Brightness | 54.1 | 92.8 | 15.8 |
| Bleaching (s) | 157 | 158 | 96 |
| Maturation time (min) | <10 | <10 | 15 |
| R_0_^*^ with mCh (nm) (4) | 5.2 | 5.5 | 4.4 |

*R_0_ calculated with refractive index of 1.4 for cellular environments.


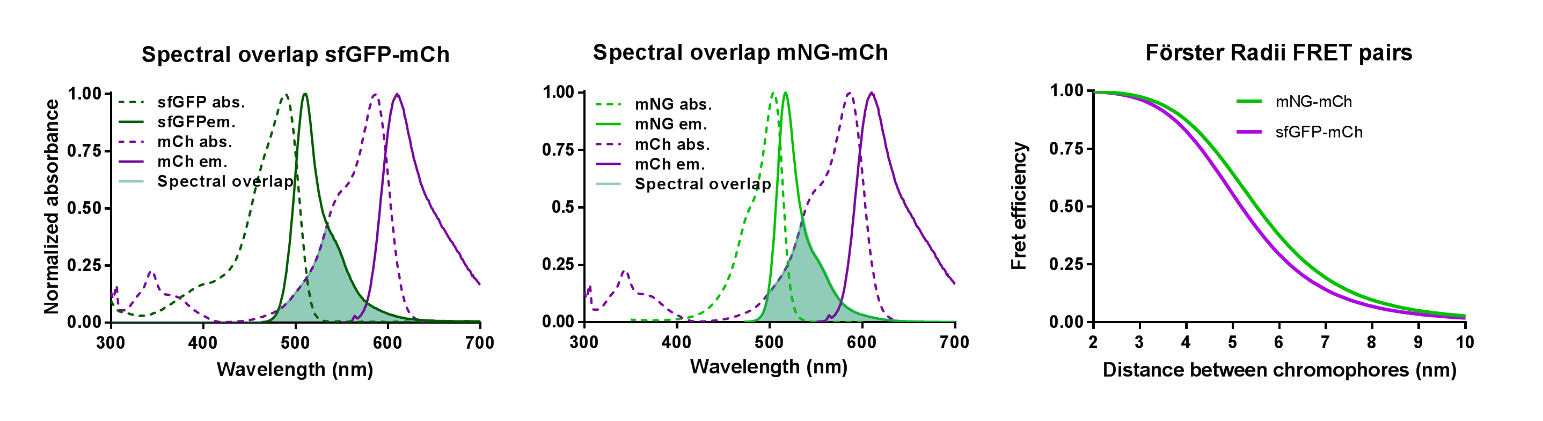


**Fig. S1.1.** Spectral overlap of mCh excitation spectrum and the emission spectrum of sfGFP (left) and mNG (middle) and the expected FRET efficiency as a function of chromophore distance for both FP-combinations (right).

**References**

1. Pédelacq J-D, Cabantous S, Tran T, Terwilliger TC, Waldo GS. 2006. Engineering and characterization of a superfolder green fluorescent protein. Nat Biotechnol 24:79–88.

2. Shaner NC, Lambert GG, Chammas A, Ni Y, Cranfill PJ, Baird MA, Sell BR, Allen JR, Day RN, Israelsson M, Davidson MW, Wang J. 2013. A bright monomeric green fluorescent protein derived from Branchiostoma lanceolatum. Nat Methods 10:407–9.

3. Shaner NC, Campbell RE, Steinbach PA, Giepmans BNG, Palmer AE, Tsien RY. 2004. Improved monomeric red, orange and yellow fluorescent proteins derived from Discosoma sp. red fluorescent protein. Nat Biotechnol 22:1567–72.

4. Foerster T. 1964. DELOCALIZED EXCITATION AND EXCITATION TRANSFER. Bulletin No. 18U.S. At. Energy Comm. FIELD Full Journal Title: Oak Ridge, TN (United States).
