# Supplementary material for: Activity-Related Conformational Changes in d,d-Carboxypeptidases Revealed by In Vivo Periplasmic Förster Resonance Energy Transfer Assay in Escherichia coli
Source: mBio. 2017 Sep 12;8(5):e01089-17. doi: 10.1128/mBio.01089-17 (PMC5596342; doi:10.1128/mBio.01089-17)
Supplement: TEXT S2 [file mbo004173468s2.docx]

## SI 2 - Toxicity of overexpressing FP fusion proteins in the periplasm

Contents

Fig. S2.1 - Expression of FP fusion proteins under rich medium growth conditions

Fig. S2.2 - Overcoming toxicity by moderate expression of FP fusion proteins

Fig. S2.3 - Expression of FP fusion proteins under minimal medium growth conditions


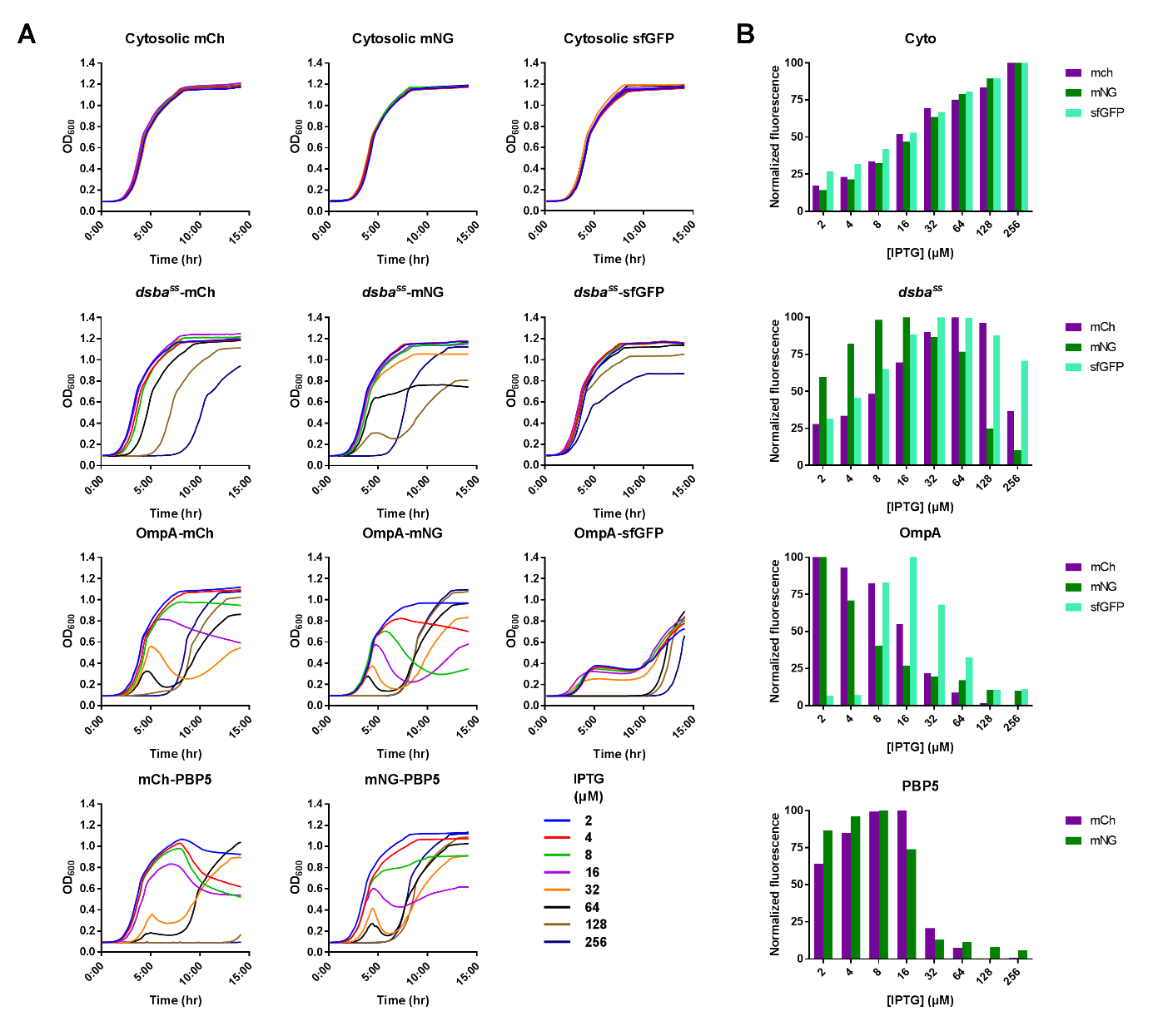


**Fig. S2.1.** Expressing exogenous periplasmic constructs can be toxic to cells and trigger selection against their expression. A) Representative growth curves of LMC500 cells in TY at 37 °C expressing cytoplasmic or periplasmic fusions of mCh, mNG and sfGFP from plasmid induced with different concentrations of IPTG. Strong induction of periplasmic FP fusions results in growth delay or arrest before continuation of regular growth B) Delayed and arrested growth samples show no or lower endpoint fluorescence correlated with the higher IPTG concentrations.

**
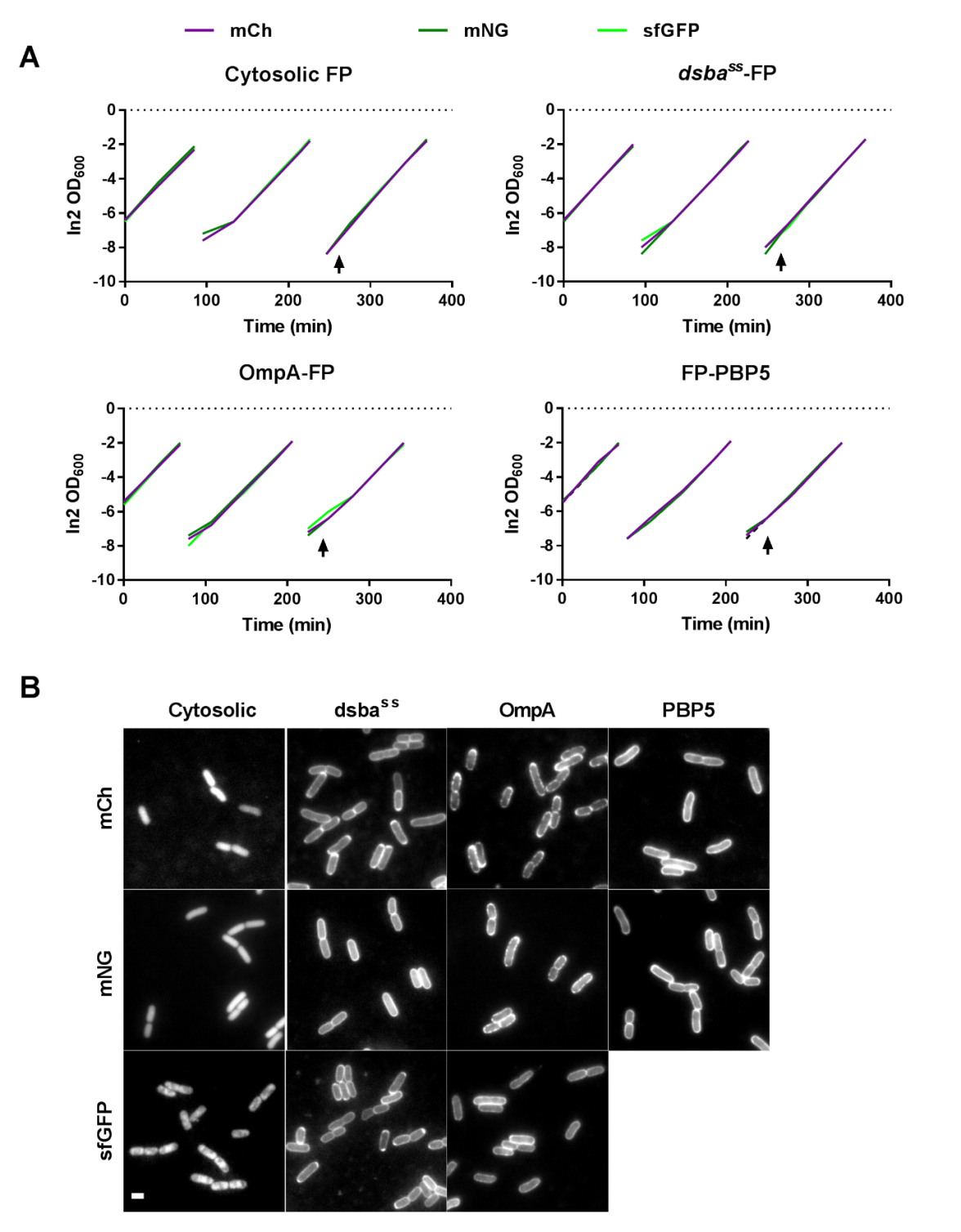
**

**Fig. S2.2.** Periplasmic expression of FPs in cells grown in rich medium at moderate induction with 15 µM IPTG does not result in cytotoxicity when OD_600_ values are kept under 0.3. A) No adverse induction effects were observed for LMC500 expressing cytoplasmic or periplasmic fusions to mCh, mNG or sfGFP at different locations in the envelope. The arrow represents the time of induction. B) Fluorescence microscopy after ~5 mass doublings revealed WT phenotypes and showed the expected localization in the cytoplasm or the periplasm depending which fusion was used. The scalebar represents 2 µm.


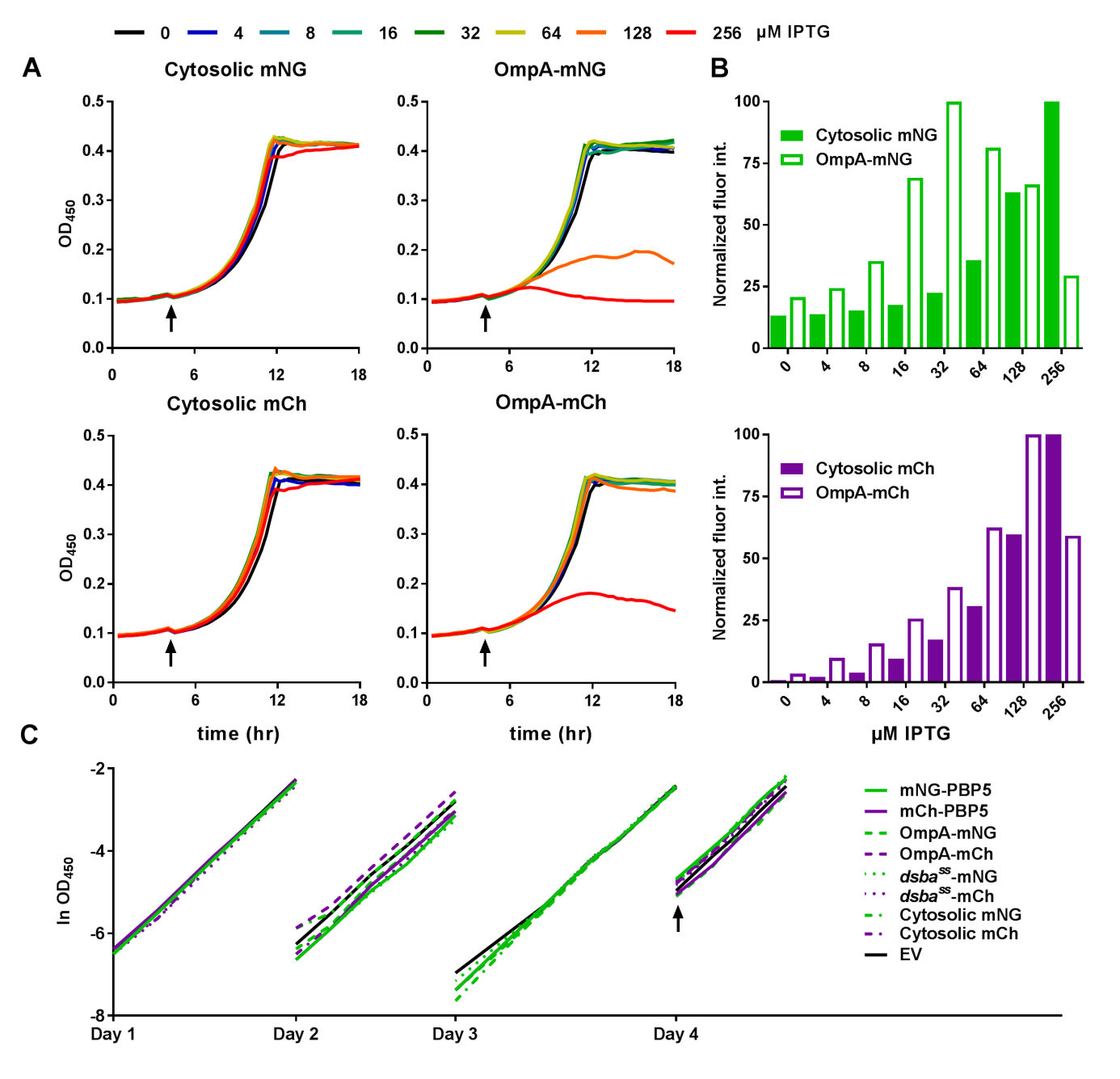


**Fig. S2.3.** Toxicity of periplasmic expression is avoided. A) Platereader growth curves of LMC500 grown in Gb1 at 28 °C expressing cytoplasmic or periplasmic fluorescent proteins from plasmid induced with 0-256 µM IPTG from the time point shown by the arrows. Growth is not adversely affected by the concentration of IPTG that induce cytoplasmic FP expression while the growth of cells expressing periplasmic OmpA-FP is impeded at the highest concentrations. B) Normalized endpoint fluorescence shows a correlation with IPTG induction and FP expression for the cytoplasmic FPs expressing cells whereas the intensity for periplasmic expressed FPs is highest for cells induced at intermediate IPTG concentrations. C) Flask culture growth curves of LMC500 cells grown to steady state in Gb1 at 28 °C expressing periplasmic FP-fusions. At day 4 the cultures were induced with 15 µM IPTG, which did not alter the growth rate. Periplasmic mNG and mCh were expressed as fusions to OmpA, the DsbA signal sequence or PBP5, cytosolic expression of mNG and mCh was diffuse and EV (Empty Vector) are cells not expressing FPs.
