# Supplementary material for: Activity-Related Conformational Changes in d,d-Carboxypeptidases Revealed by In Vivo Periplasmic Förster Resonance Energy Transfer Assay in Escherichia coli
Source: mBio. 2017 Sep 12;8(5):e01089-17. doi: 10.1128/mBio.01089-17 (PMC5596342; doi:10.1128/mBio.01089-17)
Supplement: TEXT S3 [file mbo004173468s3.docx]

## SI 3 – Periplasmic vs. Cytosolic references

Contents

Fig. S3.1 - Periplasmic and cytoplasmic mNG and mCh references are interchangeable

**Fig. S3.1.** Periplasmic and cytoplasmic mNeonGreen and mCherry references are interchangeable. Unmixing the fluorescence spectrum of the periplasmic or cytoplasmic tandem using only periplasmic or only cytoplasmic references results in the same EfA values. ns = non-significant.
