# Supplementary material for: Activity-Related Conformational Changes in d,d-Carboxypeptidases Revealed by In Vivo Periplasmic Förster Resonance Energy Transfer Assay in Escherichia coli
Source: mBio. 2017 Sep 12;8(5):e01089-17. doi: 10.1128/mBio.01089-17 (PMC5596342; doi:10.1128/mBio.01089-17)
Supplement: TEXT S4 [file mbo004173468s4.docx]

## SI 4 - Unmixing data of all spectral-based mNG-mCh FRET measurements using the fluorometer

Contents

Fig. S4.1 – Unmixing data of all spectral-based mNG-mCh FRET measurements using the fluorometer that are shown in the article


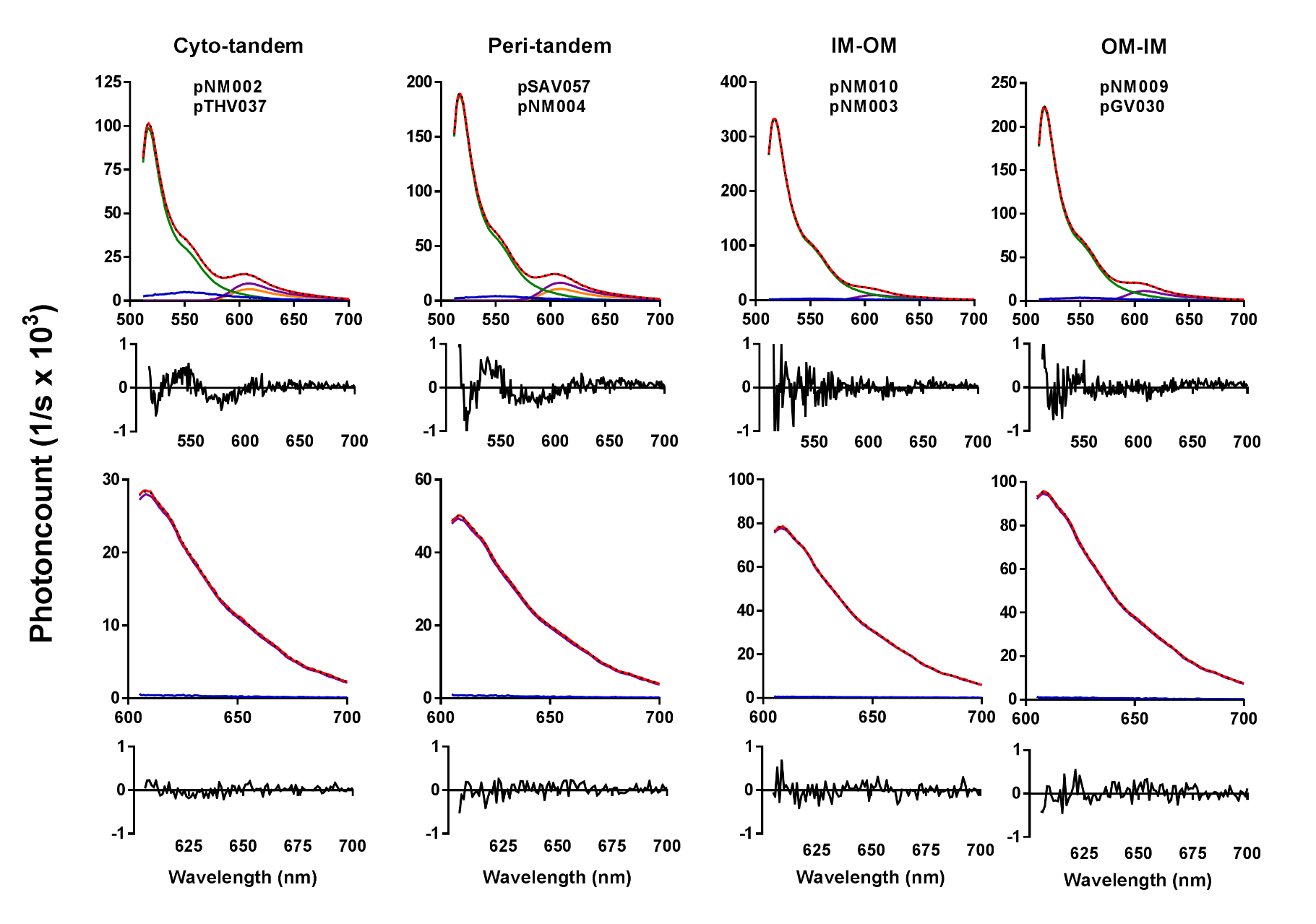


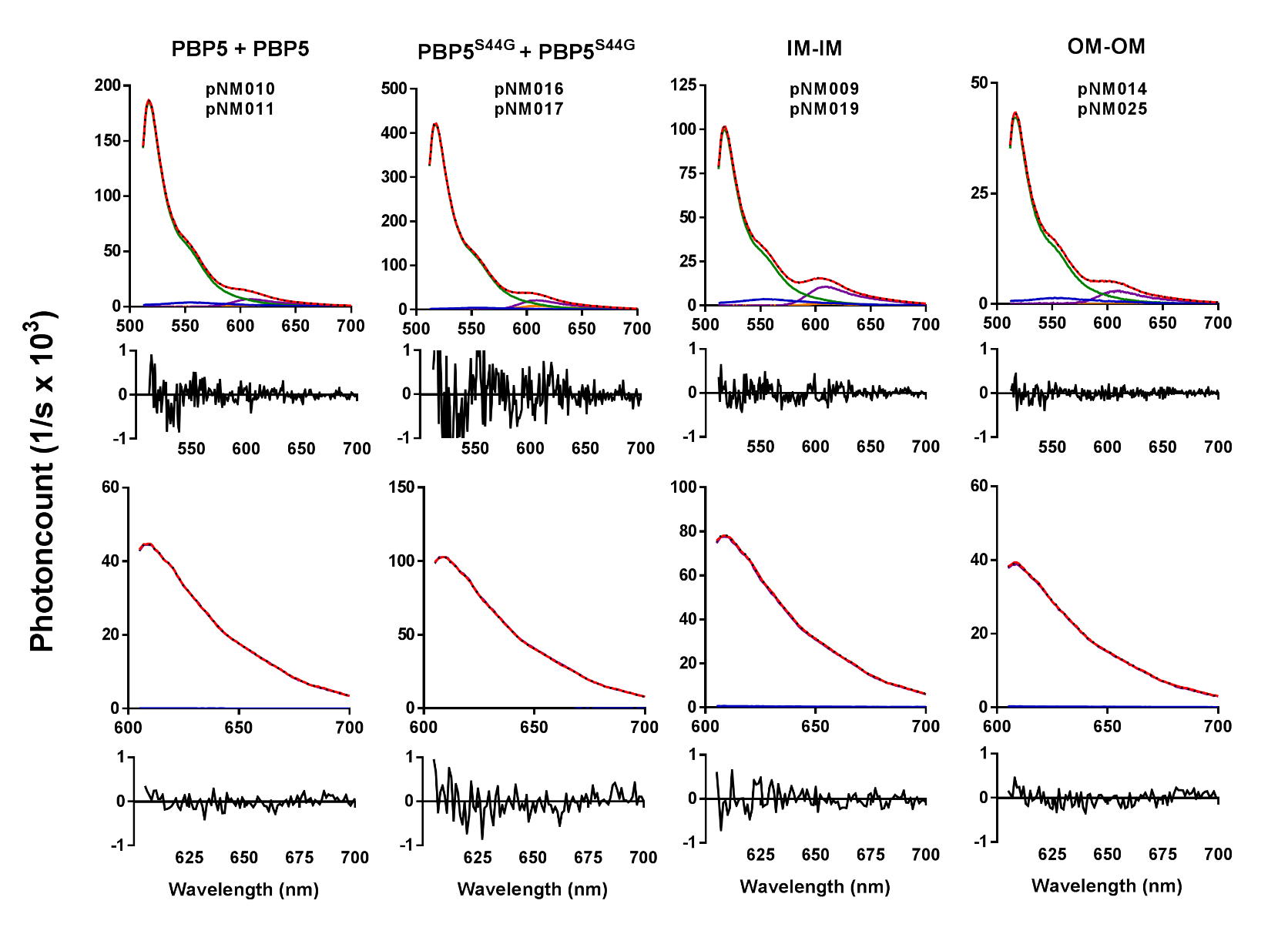

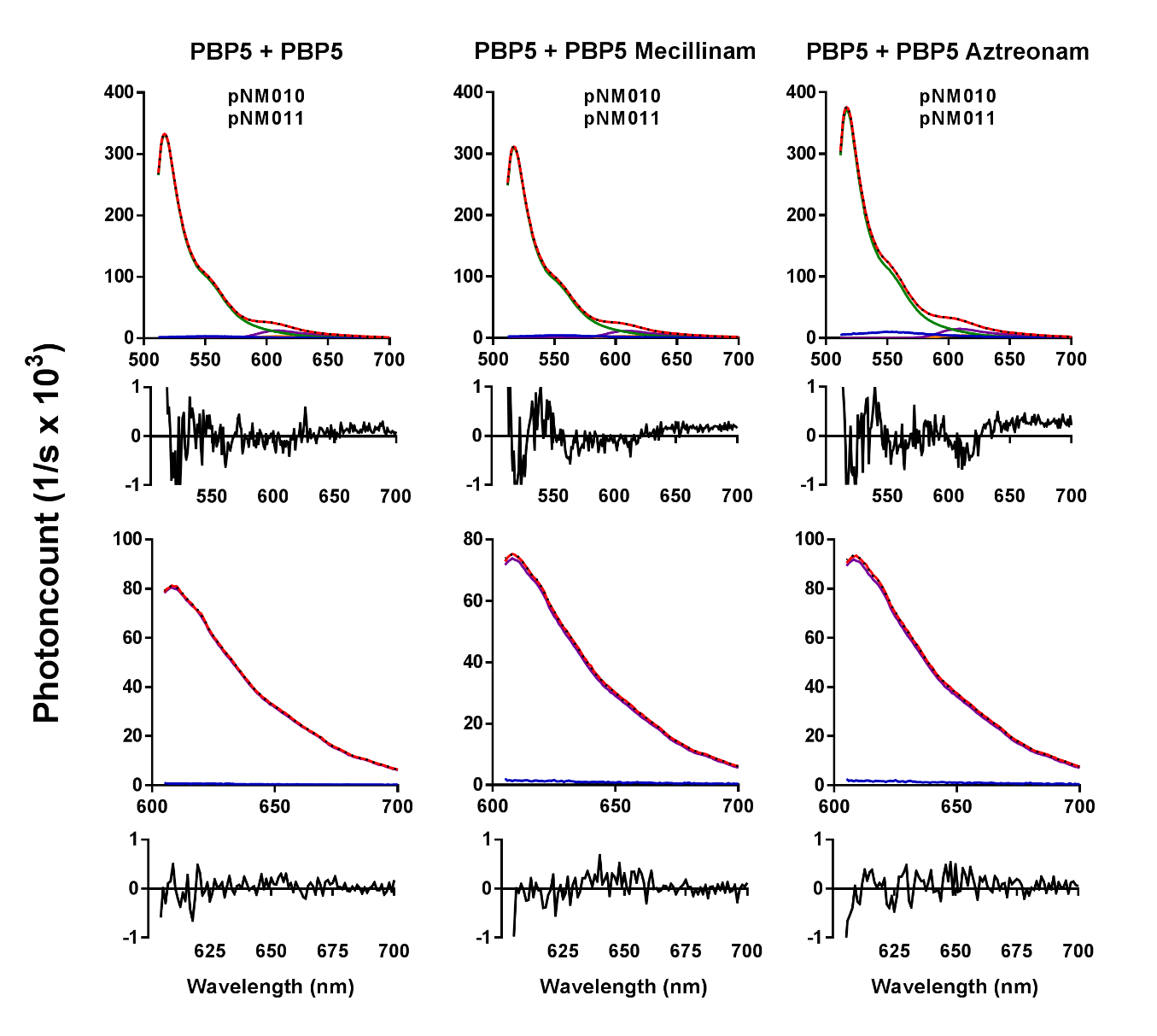


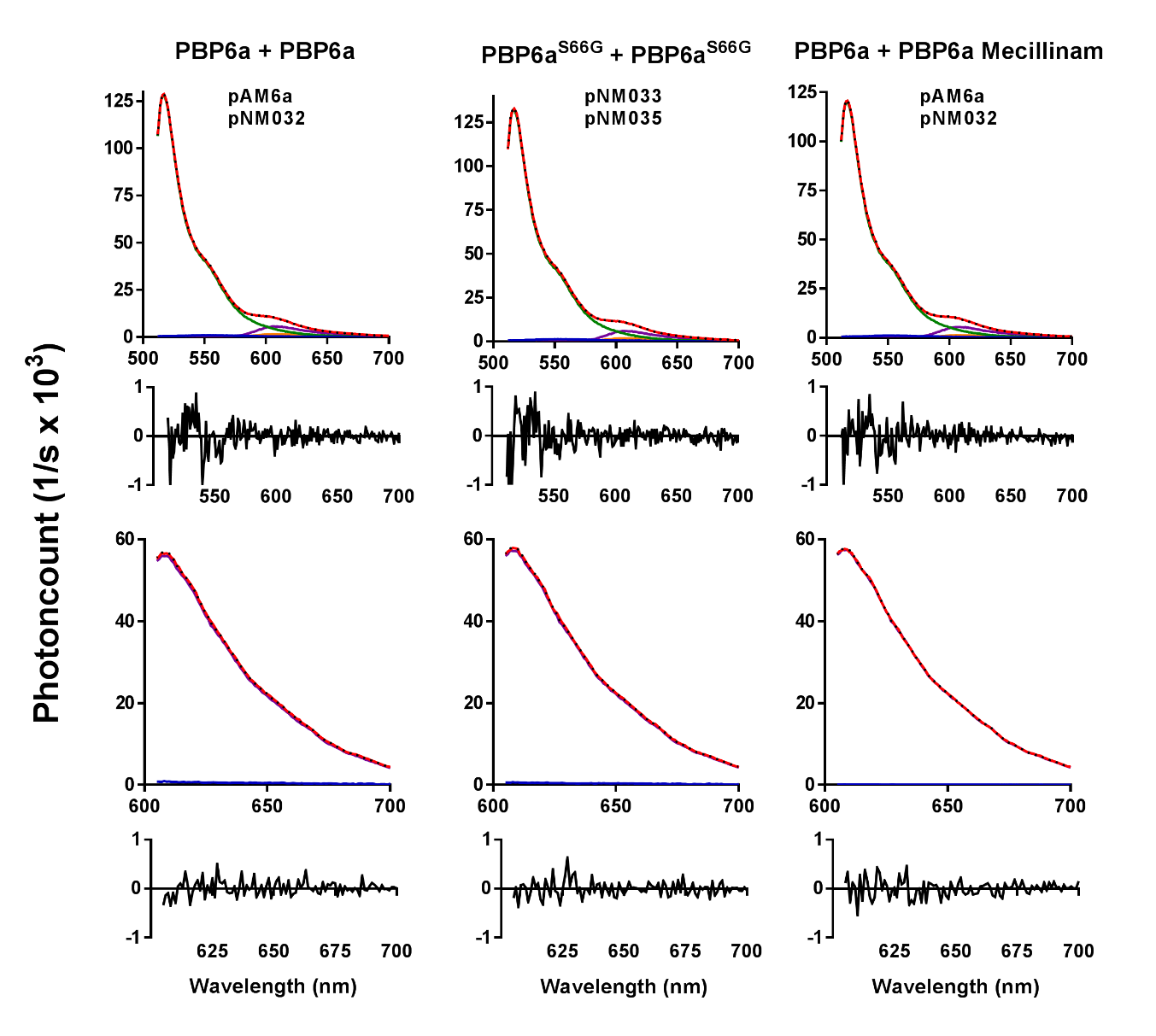

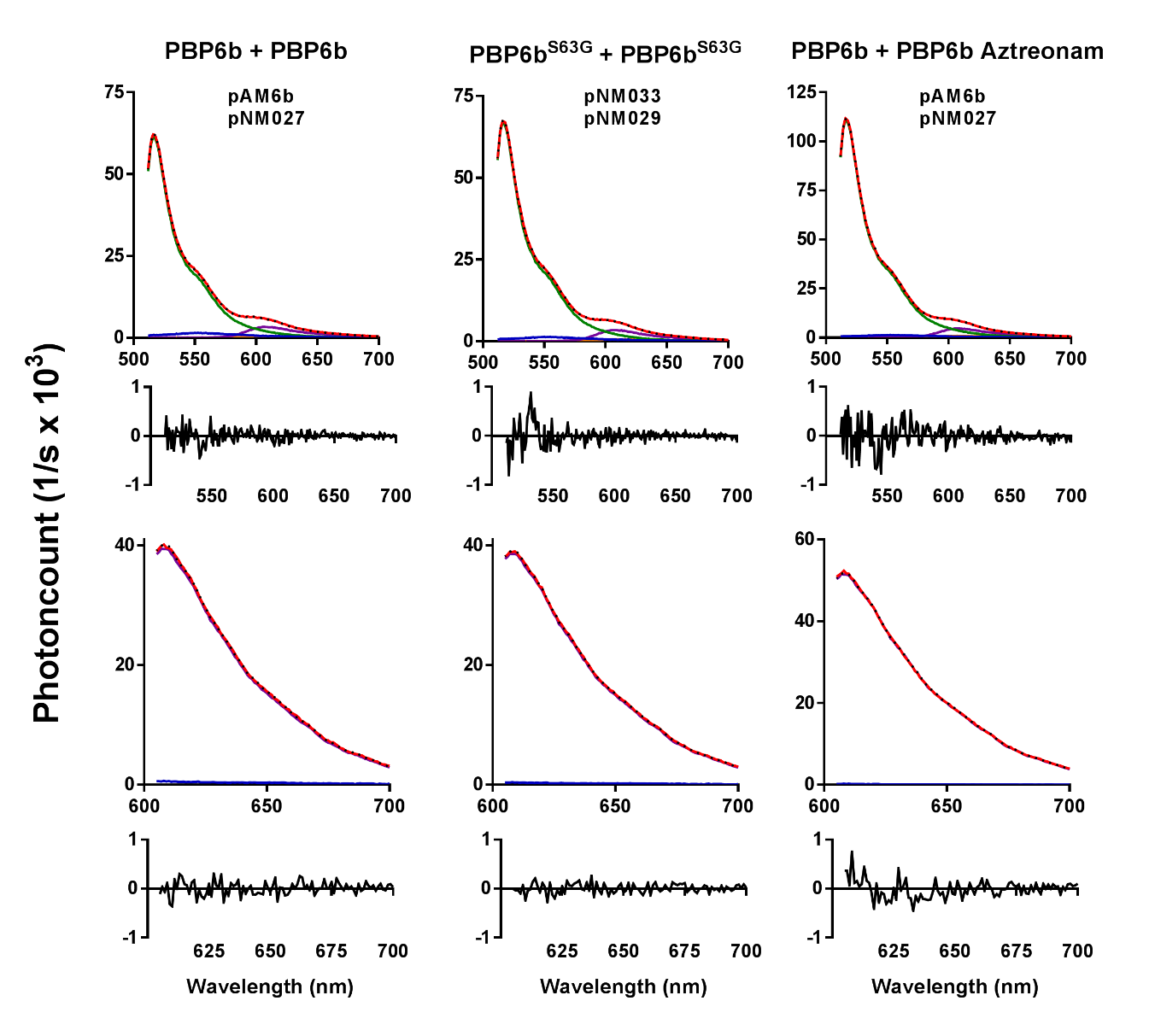


**Fig. S4.1.** Overview of the unmixing of all groups of mNG-mCh FRET samples shown in Table 1 for which the fluorescence spectra were measured using the fluorometer as described in the Material and Methods. The title above the top graph applies to graphs beneath it.
