# Supplementary material for: Activity-Related Conformational Changes in d,d-Carboxypeptidases Revealed by In Vivo Periplasmic Förster Resonance Energy Transfer Assay in Escherichia coli
Source: mBio. 2017 Sep 12;8(5):e01089-17. doi: 10.1128/mBio.01089-17 (PMC5596342; doi:10.1128/mBio.01089-17)
Supplement: TEXT S5 [file mbo004173468s5.docx]

## SI 5 – Fluorescence lifetime based FRET

Contents

Fig. S5.1 - Principle of acceptor photobleaching and periplasmic FRET by FLIM.

Table S5.1 - Fluorescence lifetimes of periplasmic mNeonGreen and FLIM-FRET values


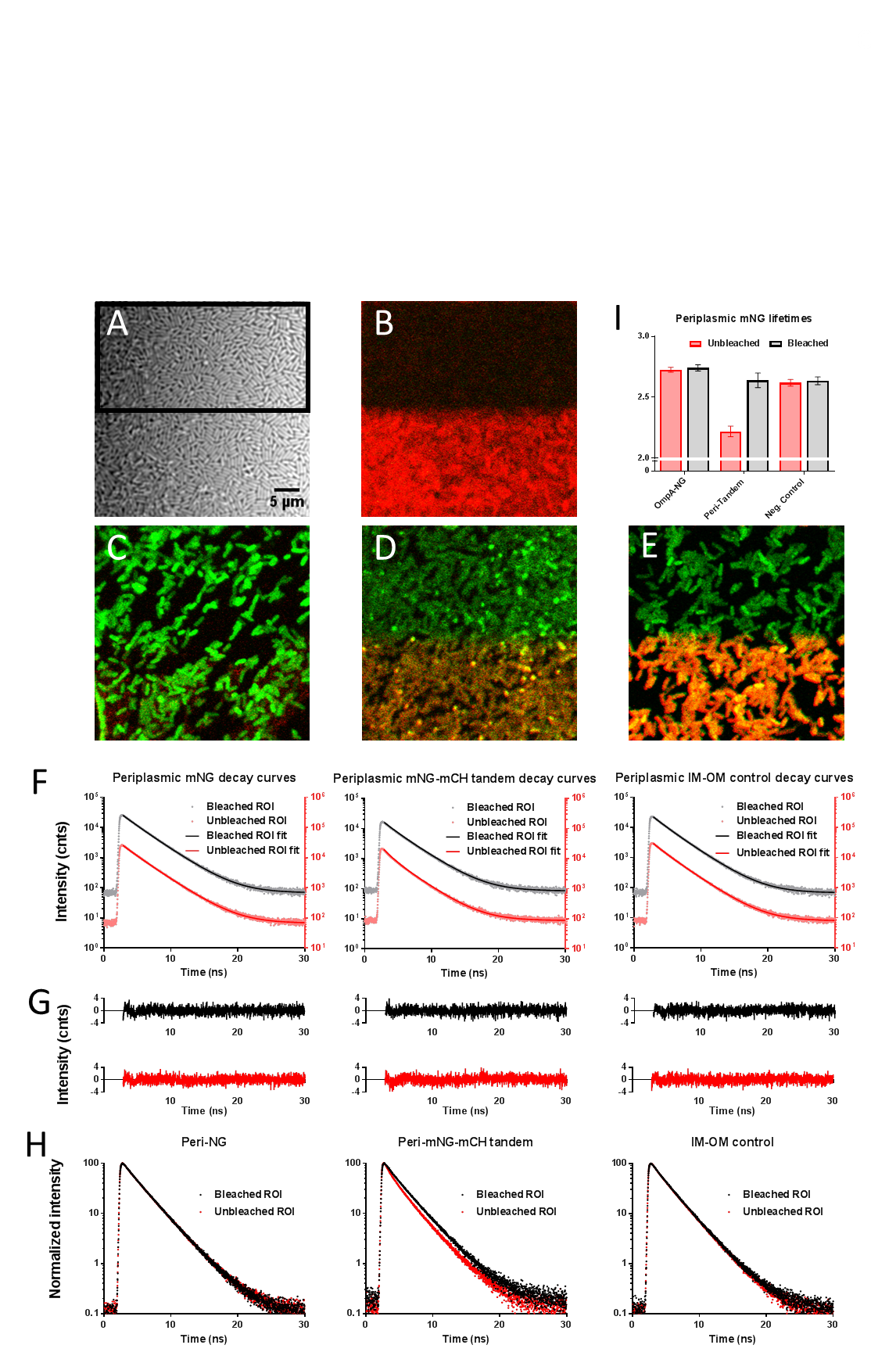


**Fig. S5.1.** Principle of FLIM acceptor photobleaching and periplasmic FRET by fluorescence lifetime measurements. A) Transmission image of immobilized cells. The top half of fields of view with cells was illuminated with a high-power laser at 561 nm to bleach all acceptor molecules. B) The mCherry signal was dramatically reduced in the bleached region of cells expressing OmpA-mCh. C) The reference donor mNG molecules expressed as OmpA-mNG are not affected by the acceptor photo bleaching and do not lose fluorescence intensity. D) The periplasmic OmpA-mNG-mCh tandem shows absence of acceptor fluorescence in the bleached region. E) The IM-OM negative control also shows absence of acceptor fluorescence in the bleached region. F) Nearly identical fluorescence decay curves of periplasmic mNG from the bleached and the unbleached region of the microscopes field of view. The left black axis and the right red axis for the bleached and unbleached mNG decay curves respectively are differently scaled to show their identical pattern while not obscuring each other. G) Residuals of periplasmic mNG fluorescence lifetimes from the bleached and unbleached fitted decay curves do not reveal a great amount of variation and suggest a good quality of measurement. H) The normalized fluorescence lifetime decay curves of the periplasmic mNG samples clearly show the steeper angle of decay for the unbleached periplasmic tandem as opposed to the bleached one which was like the mNG donor only decay curve. The mNG only or the IM-OM negative control show similar decay curves for the bleached and unbleached region. I) The calculated average lifetimes show no differences between the bleached and unbleached regions for the mNG donor reference or IM-OM control samples but the tandem does. Table 2 shows an overview of the calculated fluorescence lifetimes and their corresponding FRET efficiencies.

Table S5.1 Fluorescence lifetimes of periplasmic mNeonGreen and FLIM-FRET values

|  | **τ_amp_ Full field prebleach (ns)** | | | **τ_amp_ Unbleached ROI (ns)** | | | **τ_amp_ Bleached ROI (ns)** | | | **FRET (%)** |  |  | |  |
| --- | --- | --- | --- | --- | --- | --- | --- | --- | --- | --- | --- | --- | --- | --- |
|  |  | **± SD** | **n** |  | **± SD** | **n** |  | **± SD** | **n** |  | **± SD** | | **n** | |
| Periplasmic NG | 2.72 | 0.03 | 7 | 2.73 | 0.02 | 2 | 2.74 | 0.03 | 2 | 0.6 | 0,2 | | 2 | |
| Periplasmic tandem | 2.22 | 0.03 | 4 | 2.22 | 0.04 | 5 | 2.64 | 0.06 | 4 | 15.8 | 1.3 | | 4 | |
| IM/OM | 2.62 | 0.04 | 2 | 2.62 | 0.03 | 3 | 2.63 | 0.03 | 3 | 0.6 | 1.0 | | 3 | |
| PBP5-PBP5 | 2.47 | 0.03 | 4 | 2.49 | 0.01 | 4 | 2.59 | 0.01 | 4 | 3.6 | 0.4 | | 4 | |

ROI, region of interest; τ, fluorescence lifetime.
