# Supplementary material for: Activity-Related Conformational Changes in d,d-Carboxypeptidases Revealed by In Vivo Periplasmic Förster Resonance Energy Transfer Assay in Escherichia coli
Source: mBio. 2017 Sep 12;8(5):e01089-17. doi: 10.1128/mBio.01089-17 (PMC5596342; doi:10.1128/mBio.01089-17)
Supplement: TEXT S6 [file mbo004173468s6.docx]

## SI 6 – Platereader FRET explained, calculated EfA values and unmixing

Contents

Fig S6.1 - In vivo platereader FRET experiment and unmixing

Table S6.1 - *EfA* values from platereader experiments

Fig S6.2 - Overview of the unmixing of all fixed groups of mNG-mCh FRET

**Fig. S6** Full *in vivo* platereader FRET experiment. A) Growth curves of CS12-7 (∆*dacA*) cells grown in Gb1 at 28 °C show only small differences in doubling time for the periplasmic tandem. OD_450_ values of all well-cultures were within ~10% of each other before measuring fluorescent spectra. The arrow indicates the time point at which induction of the constructs with 20 µM IPTG was started by 1:1 dilution. B) Average normalized fluorescence spectra of periplasmic references with standard deviation error bars showing differences of maximal ~15% intensity (n=4). C) Good quality unmixing of the acceptor channel on the left and of the donor channel on the right of the average periplasmic tandem sample with average references suggests reliable calculations of sensitized emission D) Difference between the measured spectrum and the calculated spectrum with maximum unmixing differences of 56 and 72 photoncounts, respectively.

Table S6.1. *EfA* values from platereader experiments

| **mNG-mCh FRET** |  | **Fixed** |  |  | **Live** |  |
| --- | --- | --- | --- | --- | --- | --- |
| **pair** | ***EfA*** | **± SD** | **n** | ***EfA*** | **± SD** | **n** |
| periplasmic tandem | 15.2 | 1.1 | 9 | 19.1 | 0.5 | 5 |
| IM-OM | -0.4 | 0.3 | 5 | -0.3 | 0.6 | 4 |
| IM-IM | 0.3 | 0.1 | 3 | ND |  |  |
| PBP5-PBP5 | 1.7 | 0.1 | 3 | 2.1 | 1.2 | 6 |
| PBP5^S44G^-PBP5^S44G^ | 4.8 | 0.7 | 3 | ND |  |  |
| PBP6a-PBP6a | 2.6 | 0.5 | 6 | ND |  |  |
| PBP6a^S66G^-PBP6a^S66G^ | 3.3 | 0.3 | 6 | ND |  |  |
| PBP6b-PBP6b | 0.9 | 0.2 | 6 | ND |  |  |
| PBP6b^S63G^-PBP6^S63G^ | 1.5 | 0.4 | 6 | ND |  |  |

ND = Not determined


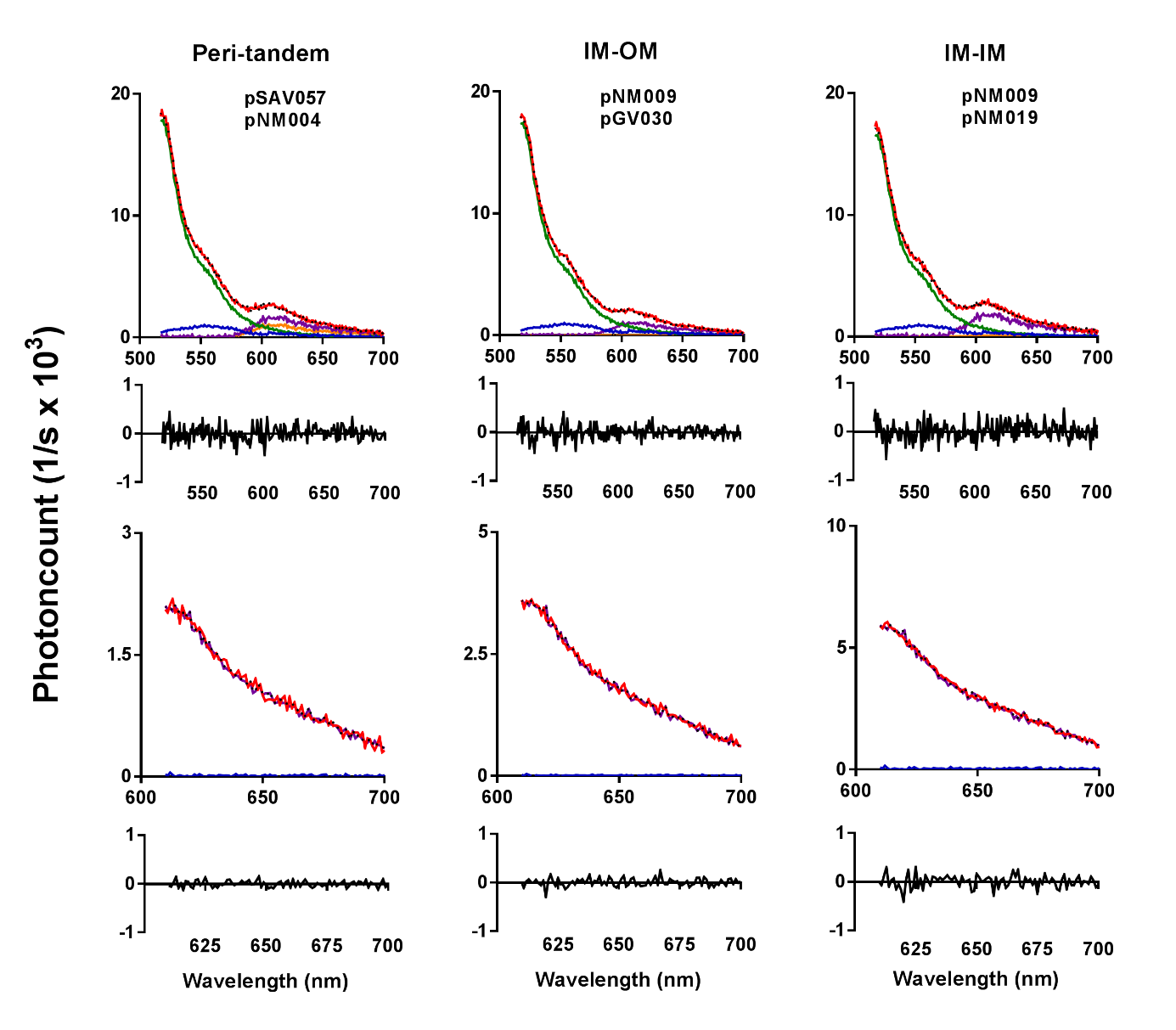


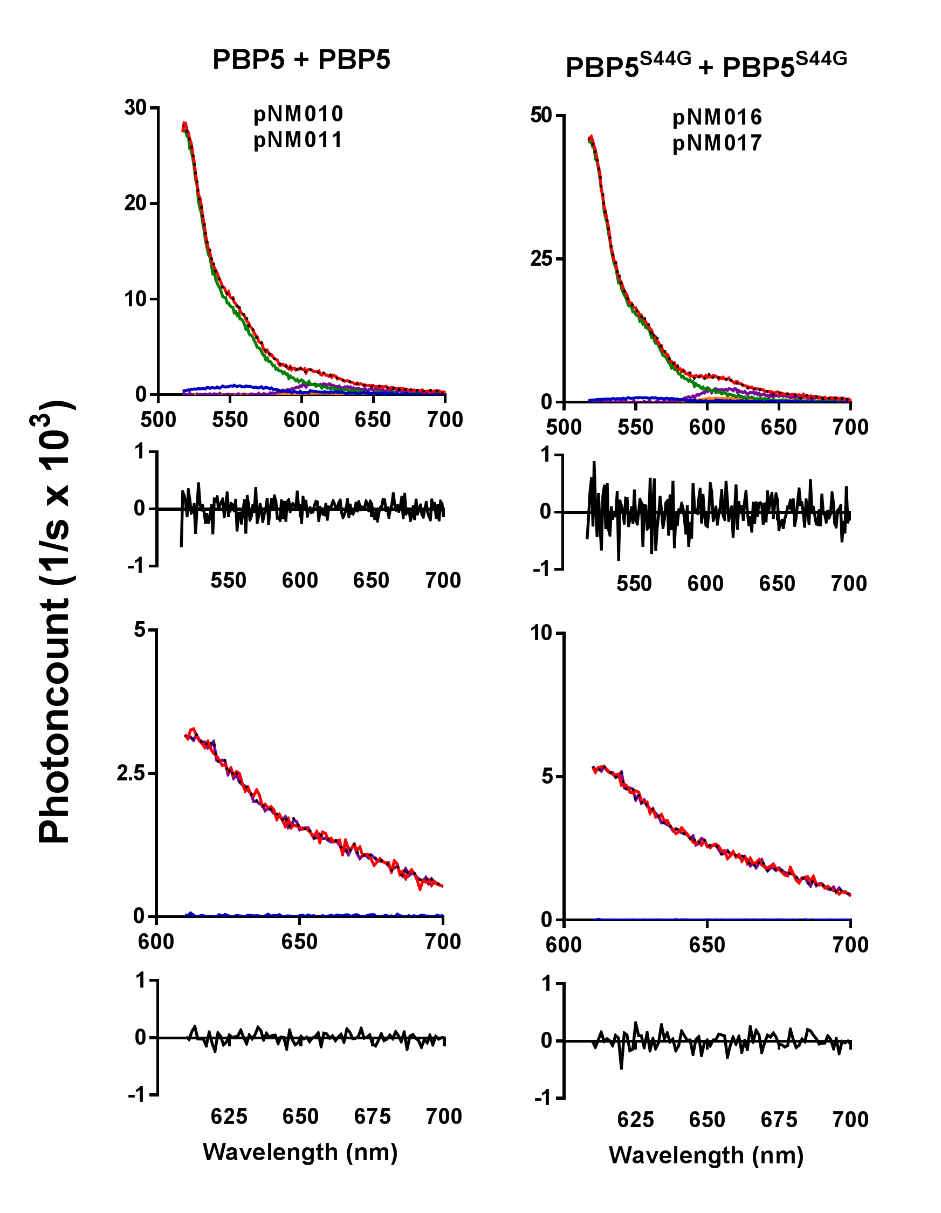

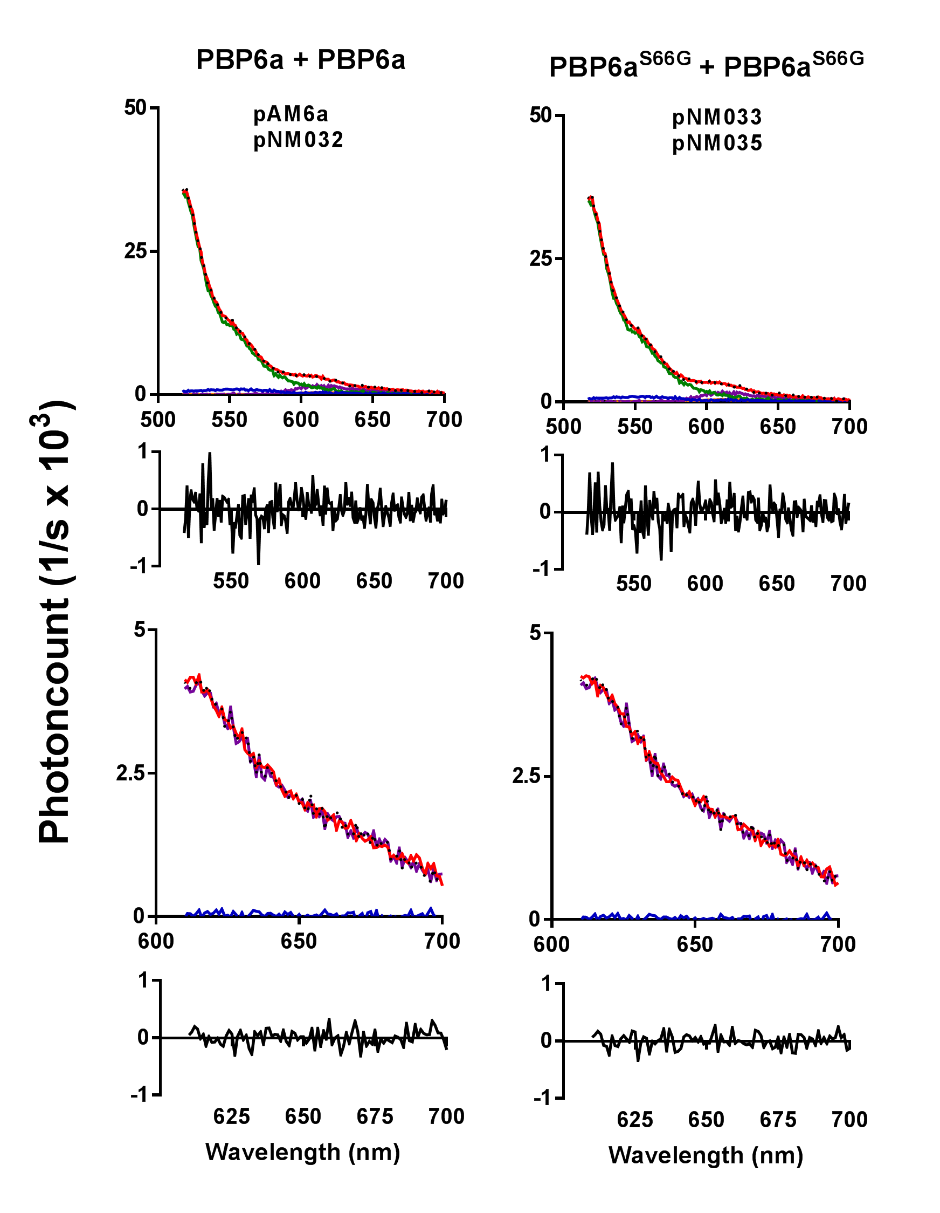

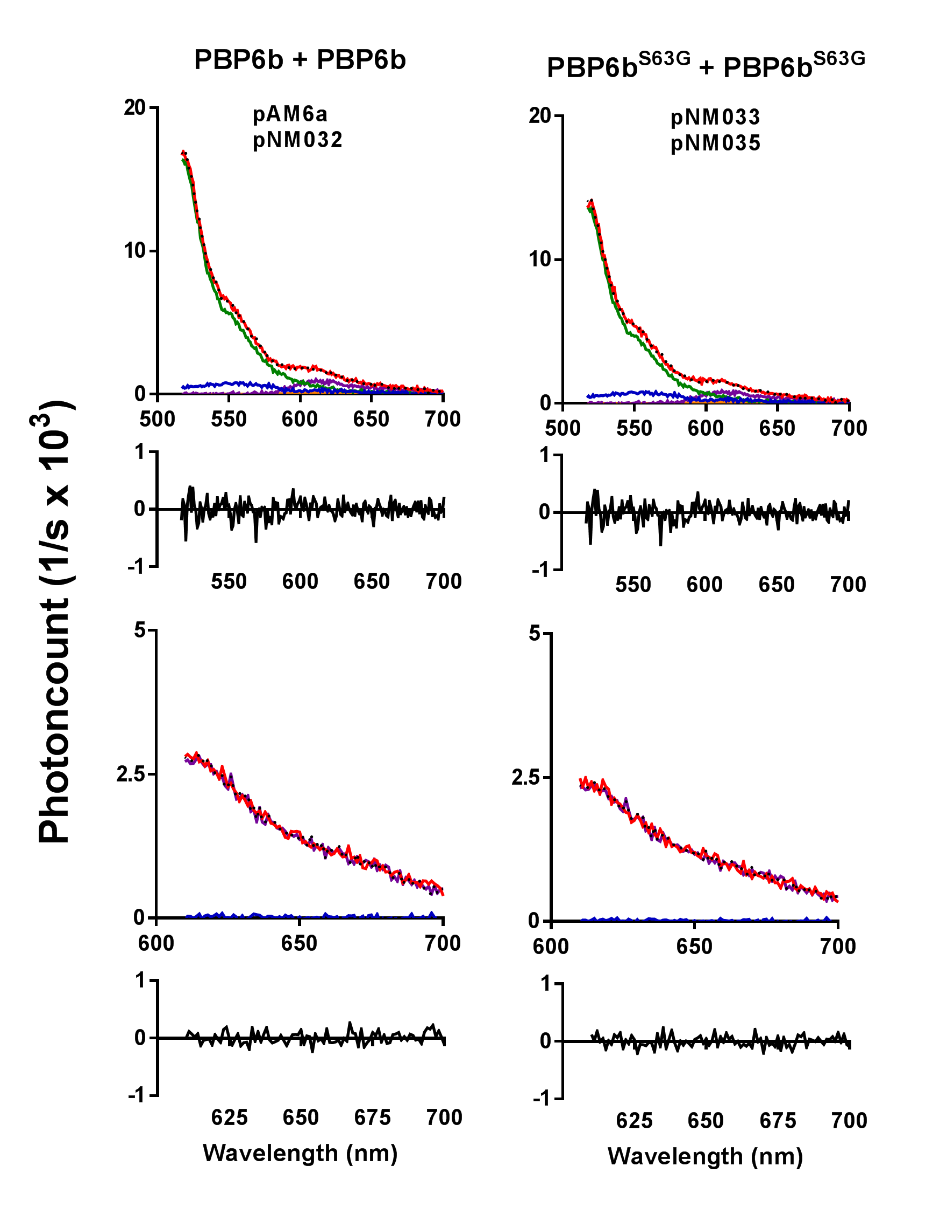


**Fig. S6.2.** Overview of the unmixing of all fixed groups of mNG-mCh FRET samples shown in Table S3 for which the fluorescence spectra were measured using the platereader as described in the Material and Methods. The title above the top graph applies to graphs beneath it.
