# Supplementary material for: Activity-Related Conformational Changes in d,d-Carboxypeptidases Revealed by In Vivo Periplasmic Förster Resonance Energy Transfer Assay in Escherichia coli
Source: mBio. 2017 Sep 12;8(5):e01089-17. doi: 10.1128/mBio.01089-17 (PMC5596342; doi:10.1128/mBio.01089-17)
Supplement: TEXT S7 [file mbo004173468s7.docx]

## SI 7 - Supplementary Material and Methods

Contents

Text S7.1 - Supplementary Material and Methods

References

## Bacterial strains and culture conditions

*Esherichia coli* K12 strains used are presented in in (SI 8). The cells were cultured in rich medium (TY: 10 g Tryptone (Bacto laboratories, Australia), 5 g yeast extract (Duchefa, Amsterdam, The Netherlands) and 5 g NaCl (Merck, Kenilworth, NJ) per liter) supplemented with 0.5% glucose (Merck) or in glucose minimal medium (Gb1: 6.33 g K_2_HPO_4_ (Merck), 2.95 g KH_2_PO_4_ (Riedel de Haen, Seelze, Germany), 1.05 g (NH_4_)_2_SO_4_ (Sigma, St. Louis, MO), 0.10 g MgSO_4_·7H_2_O (Roth, Karlsruhe, Germany), 0.28 mg FeSO_4_·7H_2_O (Sigma), 7.1 mg Ca(NO_3_)_2_·4H_2_O (Sigma), 4 mg thiamine (Sigma), and 4 g glucose per liter, pH 7.0) at 28 °C while shaking at 205 rpm. For growth in Gb1 of LMC500 and CS109 based strains 50 mg lysine (Sigma) and for growth of BW25113 and MG1655 based strains 20 mg uracil, 2 mg thymidine (Sigma), 50 mg arginine (Sigma) and 50 mg glutamine (Sigma) were added per liter. Expression of protein was induced with isopropyl β-D-1-thiogalactopyranoside (IPTG, Promega, Madison WI) as indicated. Plasmids were maintained in the strains by addition of 100 µg/ml ampicillin (Sigma) or 25 µg/ml chloramphenicol (Sigma). Growth was measured by absorbance at 600 or 450 nm with a Biochrom Libra S70 spectrophotometer (Harvard Biosciences, Holliston, MA) for TY or Gb1 cultures, respectively.

## Plasmid construction

The mNeonGreen (mNG) sequence was codon optimized for *E. coli*, flanked with 5’ *Xho*I and *Nco*I and 3’ *EcoR*I and *Hind*III restriction sites for subsequent cloning and ordered as pUC57-mNeonGreen (Genscript, Hong Kong). All expression plasmids used in this study (SI 8) were variations of the pTHV037 and pSAV057 vectors with the p*trcA*99 down promoter. All constructs were created by restriction / ligation cloning unless stated otherwise. Inserted fragments were restriction digested directly from plasmid or amplified by PCR from plasmid or the *E. coli* chromosome template and then restricted. pNM015-018, pNM028-030 and pNM033-035 were created by QuickChange site directed mutagenesis (Agilent technologies, Santa Clara, CA), A whole template was amplified by PCR using primers that contain the desired mutation. The resulting product was treated with *DpnI* to digest the methylated template plasmid. All restriction enzymes used were purchased from New England Biolabs Inc. (NEB, Ipswich, MA). All PCR amplifications for cloning were done using the High-Fidelity polymerases Phusion® or *pfu*x7 (1) as per the manufacturer’s instructions. pPP012 was created using the Gibson Assembly® Cloning Kit (NEB) as per the manufacturer’s instructions. For a complete overview of cloning strategies for each plasmid created for this study see SI 8.

## Toxicity studies

Toxicity studies were performed in transparent (Costar 3595, Corning Inc., Tewksbury, MA) or black-walled glass-bottomed (324002, Porvair Sciences Ltd., Leatherhead, UK), 96-wells plates. A 2x IPTG concentration gradient was prepared in the plate by two-fold serial dilutions in a total volume of 75 or 100 µl medium and an inoculum of the same volume of cell culture was added. Logarithmically growing TY or Gb1 cultures were pre-diluted to OD_600_ and OD_450_ values of 0.002 and 0.01 bringing the end point OD values to 0.001 and 0.005 and the total volume to 150 and 200 µl per well, respectively. The resulting IPTG end concentrations used were 256-4 and 0 µM or 128-2 and 0 µM, respectively. The plates were incubated in a prewarmed multimode platereader (BIOTEK Synergy MX, BioTek Instruments Inc., Winooski, VT) shaking at “medium” speed setting at 37 °C or 28 °C while periodically measuring OD_600_ or OD_450_ values for TY or Gb1 cultures, respectively.

## Imaging and image analysis

For imaging the cells were immobilized on 1% agarose in water slabs coated object glasses as described (2) and photographed with a Hamamatsu ORCA-Flash-4.0 (Hamamatsu, Naka-ku, Japan) CMOS camera mounted on a Nikon Eclipse Ti fluorescence microscope (Tokyo, Japan) through a 100x/N.A. 1.45 oil objective. Images were acquired using the NIS elements software. The fluorescence background was subtracted using the modal-values from the fluorescence images. Quantifications of cellular localization patterns were obtained using the ObjectJ plugin of ImageJ (3).

## Statistical analysis

All variations of mean indicated in this work are presented as standard deviations of which the sample numbers are indicated in the corresponding tables. Statistical significance was determined in Graphpad Version 7.02 software (GraphPad Software, Inc., a Jolla, CA) using a one-way ANOVA followed by a Tukey’s multiple comparisons test.

# References

1. Nørholm MH. 2010. A mutant Pfu DNA polymerase designed for advanced uracil-excision DNA engineering. BMC Biotechnol 10:21.

2. Koppelman C-M, Aarsman MEG, Postmus J, Pas E, Muijsers AO, Scheffers D-J, Nanninga N, den Blaauwen T. 2004. R174 of Escherichia coli FtsZ is involved in membrane interaction and protofilament bundling, and is essential for cell division. Mol Microbiol 51:645–57.

3. Vischer NOE, Verheul J, Postma M, van den Berg van Saparoea B, Galli E, Natale P, Gerdes K, Luirink J, Vollmer W, Vicente M, den Blaauwen T. 2015. Cell age dependent concentration of Escherichia coli divisome proteins analyzed with ImageJ and ObjectJ. Front Microbiol 6:586.
