# Supplementary material for: Activity-Related Conformational Changes in d,d-Carboxypeptidases Revealed by In Vivo Periplasmic Förster Resonance Energy Transfer Assay in Escherichia coli
Source: mBio. 2017 Sep 12;8(5):e01089-17. doi: 10.1128/mBio.01089-17 (PMC5596342; doi:10.1128/mBio.01089-17)
Supplement: TEXT S8 [file mbo004173468s8.docx]

## SI 8 – Strains, plasmids and cloning strategies

Contents

Table S8.1 - Bacterial strains used in this study

Table S8.2 - Plasmids used in this study

Table S8.3 - Cloning strategies

Table S8.4 Primers used in this study

References

Table S8.1. Bacterial strains used in this study

| **Strain** | **Relevant characteristics** | **Ref.** |
| --- | --- | --- |
| DH5α | *F^-^*, *supE44*, *hsdR17*, *recA1*, *endA1*, *gyrA96*, *thi1*, *relA1* | (1) |
| LMC500 | MC4100, *F^-^*, *araD139*, ∆(*argF-lac*)*U169*, *deoC1*, *flbB5301*,  *lysA1*, *ptsF25*, *rbsR*, *relA1*, *rpsL150* | (2) |
| BW25113 | *F^-^* Δ(*araD-araB*)*567*, *lacZ4787*(*del*)::*rrnB-3* *LAM^-^*,  *rph-1*, Δ(*rhaD-rhaB*)*568*, *hsdR514* | (3) |
| MG1655 | *F^-^*, λ^-^, *ilvG^–^*, *rfb-50*, *rph-1* | (4) |
| CS109 | *F*, *thi*, *glnV*, (*supE*), *rph-1*, *rpoS* | (5) |
| CS12-7 | CS109 Δ*dacA* | (5) |

Table S8.2. Plasmids used in this study

| **Plasmid** | **Full Name** | **Relevant characteristics** | **Reference** |
| --- | --- | --- | --- |
| pTHV037 |  | Basic expression vector p*trc*99A derived, differing from in its with  different -35 promotor region (TTGACA-TTTACA). pBR322 origin,  AmpR | (6) |
| pSAV057 |  | Basic expression vector p*trc*99A derived, with different -35  promotor Region (TTGACA-TTTACA) p15 origin, catR | (7) |
| pUC57-mNG^EC^ |  | *E.coli* codon optimized mNeonGreen (mNG^EC^) | This work |
| pNM001 | pSAV057-mNG^EC^ | Cytoplasmic mNG^EC^ reference | This work |
| pSAV047 | pTHV037-mCh | Cytoplasmic mCh reference | (7) |
| pNM002 | pSAV057-mNG^EC^-mCh | Cytoplasmic tandem | This work |
| pNM003 | pTHV037-Ompa-SA1-177-(SA-1)-LEDPPAEL-mNG^EC^ | OM bound OmpA-mNG^EC^ reference | This work |
| pGV030 | pTHV037 OmpA-177-(SA-1)-LEDPPAEF-mCh | OM bound OmpA-mCh reference | (8) |
| pNM004 | pTHV037-OmpA-SA1-177-(SA-1)-LEDPPAEL-NG^EC^-mCh | OM bound OmpA-mNG^EC^-mCh tandem | This work |
| pNM013 | pSAV057-OmpA-SA1-177-(SA-1)-LEDPPAEF-mCh | OM bound OmpA-mCh reference | This work |
| pNM014 | pSAV057-Ompa-SA1-177-(SA-1)-LEDPPAEL-mNG^EC^ | OM bound OmpA-mNG^EC^ reference | This work |
| pNM005 | pTHV037-OmpA-SA1-177-(SA-1)-LEDPPAEF-sfGFP | OM bound sfGFP | This work |
| pNM006 | pTHV037-DsbA^ss^-mNG^EC^ | Unbound periplasmic mNG^EC^ | This work |
| pNM007 | pTHV037-DsbA^ss^-mNG^EC^-mCh | Unbound periplasmic mNG^EC^-mCh tandem | This work |
| pNM008 | pSAV057-DsbA^ss^-mNG^EC^ | Unbound periplasmic mNG^EC^ | This work |
| pNM009 | pSAV057-DsbA^ss^-LEGPAGL-mNG^EC^-EFGS-∆1-17-PBP5 | IM bound mNG^EC^-PBP5 | This work |
| pNM010 | pSAV057-DsbA^ss^-LEGPAGL-mCh-EFSGRS-∆1-17-PBP5 | IM bound mCh-PBP5 | This work |
| pNM011 | pTHV037-DsbA^ss^-LEGPAGL-mNG^EC^-EFGS-∆1-17-PBP5 | IM bound mNG^EC^-PBP5 | This work |
| pNM012 | pTHV037-DsbA^ss^-LEGPAGL-mCh-EFSGRS-∆1-17-PBP5 | IM bound mCh-PBP5 | This work |
| pNM015 | pSAV057-DsbA^ss^-LEGPAGL-mNG^EC^-EFGS-∆1-17-PBP5-S44G | IM bound mNG^EC^-PBP5 S44G mutant | This work |
| pNM016 | pSAV057-DsbA^ss^-LEGPAGL-mCh-EFSGRS-∆1-17-PBP5-S44G | IM bound mCh-PBP5 S44G mutant | This work |
| pNM017 | pTHV037-DsbA^ss^-LEGPAGL-mNG^EC^-EFGS-∆1-17-PBP5-S44G | IM bound mNG^EC^-PBP5 S44G mutant | This work |
| pNM018 | pTHV037-DsbA^ss^-LEGPAGL-mCh-EFSGRS-∆1-17-PBP5-S44G | IM bound mCh-PBP5 S44G mutant | This work |
| pNM019 | pTHV037-FtsN-GGSEF-mCh | IM bound FtsN-mCh, crowding control, 5' GTG start codon changed  to ATG, L315I | This work |
| pNM021 | pSAV057-FtsN-GGSEF-mCh | IM bound FtsN-mCh, crowding control, 5' GTG start codon changed  to ATG, L315I | This work |
| pNM025 | pTHV037-MG-TolC-mCh | OM bound TolC-mCh | This work |
| pPP012 | pSAV057-TolC-mNG^EC^ | OM bound TolC-mNG^EC^ | This work |
| pNM026 | pTHV037-DsbA^ss^-LEGPAGL-mCh-EFSGRS-∆1-20-PBP6b | IM bound mCh-PBP6b | This work |
| pNM027 | pTHV037-DsbA^ss^-LEGPAGL-mNG-EFSGRS-∆1-20-PBP6b | IM bound mNG-PBP6b | This work |
| pAM6b | pSAV057-DsbA^ss^ -LEGPAGL-mCh-EFSGRS-∆1-20-PBP6b | IM bound mCh-PBP6b | This work |
| pNM028 | pTHV037-DsbA^ss^ -LEGPAGL-mCh-EFSGRS-∆1-20-PBP6b-S63G | IM bound mCh-PBP6b S63G mutant | This work |
| pNM029 | pTHV037-DsbA^ss^ -LEGPAGL-mNG^EC^-EFSGRS-∆1-20-PBP6b-S63G | IM bound mNG-PBP6b S63G mutant | This work |
| pNM030 | pSAV057-DsbA^ss^-LEGPAGL-mCh-EFSGRS-∆1-20-PBP6b-S63G | IM bound mCh-PBP6b S63G mutant | This work |
| pNM031 | pTHV037-DsbA^ss^-LEGPAGL-mCh-EFSGRS-∆1-27-PBP6a | IM bound mCh-PBP6a | This work |
| pNM032 | pTHV037-DsbA^ss^ -LEGPAGL-mNG^EC^-EFSGRS-∆1-27-PBP6a | IM bound mNG-PBP6a | This work |
| pAM6a | pSAV057-DsbA^ss^-LEGPAGL-mCh-EFSGRS-∆1-27-PBP6a | IM bound mCh-PBP6a | This work |
| pNM033 | pTHV037-DsbA^ss^ -LEGPAGL-mCh-EFSGRS-∆1-27-PBP6a-S66G | IM bound mCh-PBP6a S66G mutant | This work |
| pNM034 | pTHV037-DsbA^ss^-LEGPAGL-mNG-EFSGRS-∆1-27-PBP6a-S66G | IM bound mNG-PBP6a S66G mutant | This work |
| pNM035 | pSAV057-DsbA^ss^-LEGPAGL-mCh-EFSGRS-∆1-27-PBP6a-S66G | IM bound mCh-PBP6a S66G mutant | This work |
| pDSK001 | pSAV057-DsbA^ss^-sfGFP | Unbound periplasmic sfGFP | This work |
| pRP081 | pSAV057-DsbA^ss^-mCh | Unbound periplasmic mCh | This work |
| pRRC01 | pTHV037-DsbA^ss^-mCh | Unbound periplasmic mCh | This work |
| pRRC02 | pTHV037-DsbA^ss^-mCh-PBP5 | IM bound mCh-PBP5 | This work |
| pRRC03 | pTHV037-DsbA^ss^-mCh-sfGFP | Unbound periplasmic mCh-sfGFP tandem | This work |
| pRRC04 | pSAV057-DsbA^ss^-sfGFP-PBP5 | IM bound sfGFP-PBP5 | This work |
| pRRC05 | pSAV057-DsbA^ss^-sfGFP-mCh | IM bound mCh-PBP5 | This work |

Table S8.3. Cloning strategies

| **Plasmid** | **Vector backbone** | **primers** | **RE1** | **RE2** | **Insert** | **Template** | **primers** | **RE1** | **RE2** |
| --- | --- | --- | --- | --- | --- | --- | --- | --- | --- |
| pNM001 | pSAV057 | - | NcoI | HindIII | mNG | pUC57-NG | - | NcoI | HindIII |
| pNM002 | pNM001 | - | EcoRI | HindIII | mCh | pGV030 | - | EcoRI | HindIII |
| pNM003 | pGV030 | - | EcoRI | HindIII | mNG | pUC57-NG | 39 / 40 | MfeI | HindIII |
| pNM004 | pNM003 | - | EcoRI | HindIII | mCh | pGV030 | - | EcoRI | HindIII |
| pNM005 | pGV030 | - | EcoRI | HindIII | sfGFP | pSAV-YbgF-sfGFP* | 13 / 14 | EcoRI | HindIII |
| pNM006 | pRRC01 | - | XhoI | HindIII | mNG | pUC57-NG | - | XhoI | HindIII |
| pNM007 | pNM006 | - | EcoRI | HindIII | mCh | pGV030 | - | EcoRI | HindIII |
| pNM008 | pRP081 | - | XhoI | HindIII | mNG | pUC57-NG | - | XhoI | HindIII |
| pNM009 | pRRC04 | - | XhoI | BamHI | mNG | pNM001 | 73 / 74 | XhoI | BamHI |
| pNM010 | pRRC04 | - | XhoI | BamHI | mCH | pSAV47 | 75 / 76 | XhoI | BamHI |
| pNM011 | pRRC02 | - | XhoI | BamHI | mNG | pNM001 | 73 / 74 | XhoI | BamHI |
| pNM012 | pRRC02 | - | XhoI | BamHI | mCH | pSAV47 | 75 / 76 | XhoI | BamHI |
| pNM013 | pNM001 | - | NcoI | HIndIII | OmpA-mCh | pGV030 | - | NcoI | HIndIII |
| pNM014 | pNM001 | - | NcoI | HIndIII | OmpA-mNG | pNM003 | - | NcoI | HIndIII |
| pNM015 | pNM009 | 173 / 174 | - | - | - | - | - | - | - |
| pNM016 | pNM010 | 173 / 174 | - | - | - | - | - | - | - |
| pNM017 | pNM011 | 173 / 174 | - | - | - | - | - | - | - |
| pNM018 | pNM012 | 173 / 174 | - | - | - | - | - | - | - |
| pNM019 | pGV030 | - | NcoI | EcoRI | FtsN | chromosome | 124 / 125 | NcoI | EcoRI |
| pNM021 | pNM001 | - | NcoI | BsrGI | FtsN-mCh | pNM019 | - | NcoI | BsrGI |
| pNM025 | pGV030 | - | NcoI | EcoRI | TolC | Chromosome | 211 / 212 | NcoI | EcoRI |
| pPP012 | pNM001 | Vec.rev / for | - | - | TolC | Chromosome | Frag.rev / for | - | - |
| pNM026 | pNM012 | - | EcoRI | HindIII | PBP6b | Chromosome | 180 / 181 | EcoRI | HindIII |
| pNM027 | pNM026 | - | Xhoi | EcoRI | mNG | pNM011 | 73 / 137 | Xhoi | EcoRI |
| pAM6b | pSAV057 | - | EcoRI | HindIII | PBP6b | pNM026 | 180 / 181 | EcoRI | HindIII |
| pNM028 | pNM026 | 219 / 220 | - | - | - | - | - | - | - |
| pNM029 | pNM028 | - | XhoI | EcoRI | mNG | pNM011 | 73 / 137 | XhoI | EcoRI |
| pNM030 | pAM6b | 219 / 220 | - | - | - | - | - | - | - |
| pNM031 | pNM012 | - | EcoRI | HindIII | PBP6a | Chromosome | 178 / 179 | EcoRI | HindIII |
| pNM032 | pNM031 | - | XhoI | EcoRI | mNG | pNM011 | 73 / 137 | XhoI | EcoRI |
| pAM6a | pSAV057 | - | EcoRI | HindIII | PBP6a | pNM031 | 178 / 179 | EcoRI | HindIII |
| pNM033 | pNM031 | 217 / 218 | - | - | - | - | - | - | - |
| pNM034 | pNM033 | - | XhoI | EcoRI | mNG | pNM011 | 73 / 137 | XhoI | EcoRI |
| pNM035 | pAM6a | 217 / 218 | - | - | - | - | - | - | - |

* This plasmid was a kind gift from Andrew Gray.

Table S8.4. Primers used in this study

| **#** | **name** | **5' → 3'** | **Characteristics of primer combination** |
| --- | --- | --- | --- |
| **39** | MfeI NeonGreenF | tttttt*caattg*ATGGTGAGCAAGGGCGA | Introduces MfeI site |
| **40** | NeonGreen HindIIIR | ggggg*AAGCTT*TAGAATTCCTTGTACAGCT | HindIII on template |
| **13** | OmpA-sfGFP cloning | tttt*gaattC*CGTAAAGGTGAAGAACTGTTCACCGGT | Introduces EcoRI site |
| **14** | OmpA-sfGFP cloning | GTTCGGGCC*CAAGCTT*GCATGC | HindIII on template |
| **73** | 73-XhoI-link-NG | ggggg*ctcgag*ggtccggctggtctgATGGTGAGCAAGGGCGAG | Introduces XhoI site and LEGPAGL linker |
| **74** | 74-NG-nostop-BamHI | atatat*ggatcc*GAATTCCTTGTACAGCTCGTCC | Excludes stop codon and introduces BamHI site |
| **75** | 75-XhoI-link-mCherry | ggggg*ctcgag*ggtccggctggtctgATGGTGAGCAAGGGCGAGGAG | Introduces XhoI site and LEGPAGL linker |
| **76** | 76mCh-nostop-BamHI | atatat*ggatcc*TCTAGAGAATTCCTTGTACAGCTCG | Excludes stop codon and introduces BamHI site |
| **77** | pTHV/pSAV-seq-F | GCACTCCCGTTCTGGATAATG | Sequencing primers for pTHV and pSAV based |
| **78** | pTHV/pSAV-seq-R | TTATCAGACCGCTTCTGCG | plasmids |
| **137** | 137-FP-nostp-EcoRI | cccccgaattcCTTGTACAGCTCGTCCATG | Excludes stop codon and introduces EcoRI site |
| **173** | 173-PBP5-S44G-F | GATGTCCGCC**g**GGATCCTGCCgGCCTGACCAAAATGATGA | Introduces S44G mutation in PBP5 |
| **174** | 174-PBP5-S44G-R | TCATCATTTTGGTCAGGCcGGCAGGATC**c**CGGCGGACATC | And adds silent mutation for additional BamHI site |
| **124** | 124-NcoI-FtsN | ggggg*cc****a****TG*GCACAACGAGATTATG | Introduces NcoI site, exchange GTG to ATG |
| **125** | 125-FtsN-nostop-EcoRI | ccccc*gaattc*gctaccaccACCACCtGCtGCGAtCCGAATGCAGTTTGTAT | Excludes stop codon, adds GGS linker and MfeI site |
|  | Vector.rev | GGTCTGTTTCCTGTGTGAAATTGTTATCCGC | Amplification pNM001 vector part |
|  | Vector.for | ATGGTGAGCAAGGGCGAGGAG | for Gibson assembly |
|  | Fragment.rev | CGCCCTTGCTCACCATgttacggaaagggttatgaccgttactggt | Amplification *E. coli* TolC |
|  | Fragment.for | cgcccttgctcaccatGTTACGGAAAGGGTTATGACCGTTACTGGT | for Gibson assembly |
| **177** | 177-PBP5-seq-R | CAGACCATGTACCGTCTGGAAGTG | Internal PBP5 sequencing primer |
| **178** | 178-ecoRI-PBP6a | ggaga*gaattc*tctagaggatCGGCGGAACAAACCGTTGAAGCGCC | Introduces EcoRI site |
| **179** | 179-PBP6a-HindIII | ggagg*aagctt*TTAAGAGAACCAGCTGCCGAACC | Introduces HindIII site |
| **180** | 180-EcoRI-PBP6b | ggaga*gaattc*tctagaggatCGGCGGAAAACATTCCTTTTTCACCTCAGCCTC | Introduces EcoRI site |
| **181** | 181-PBP6b-HindIII | ggagg*aagctt*TCAGGCCTTATGGTGGAAATAATCACTCAGGC | Introduces HindIII site |
| **211** | 211-NcoI-TolC | gggaa*ccatg*ggcATGAAGAAATTGCTCCCCATTCTTATCGGCCTG | Introduces NcoI site |
| **212** | 212-TolC-EcoRI | gggcgc*gaattc*GTTACGGAAAGGGTTATGACCGTTACTGGTGG | Excludes stop codon, adds MfeI site |
| **217** | 217-PBP6A-S66G-F | GCGGATGAGAAACTGGA**c**CCCGCG**g**GCCTGACTAAAATCATGACCAGCTATGTGG | Introduces S66G mutation in PBP6a and an |
| **218** | 218-PBP6A-S66G-R | CCACATAGCTGGTCATGATTTTAGTCAGGC**c**CGCGGG**g**TCCAGTTTCTCATCCGC | silent mutation to take out a BamHI site |
| **219** | 219-PBP6b-S63G-F | GAGCATCAACAGCGCAATCCCGCC**g**GCCTGACAAAGCTGATGACGGGTTATGTCG | Introduces S63G mutation in PBP6b |
| **220** | 220-PBP6b-S63G-R | CGACATAACCCGTCATCAGCTTTGTCAGGC**c**GGCGGGATTGCGCTGTTGATGCTC |  |
| **224** | 224-PBP6a-seq-F | GCCAGCGCAATACAGGCGTCATTACCGGAC | Internal PBP6a sequencing primer |
| **225** | 225-PBP6b-seq-R | GTCAGCCAGAGCAACACAAGCGTCATTTCC | Internal PBP6b sequencing primer |

Uppercase bases anneal to template DNA, *italics* signify restriction sites and **bold** underscores signify mutation

References

1. Bethesda Research Laboratories. 1986. E. coli DH5 alpha competent cells. Focus - Bethesda Res Lab 8:9.

2. Taschner PE, Huls PG, Pas E, Woldringh CL. 1988. Division behavior and shape changes in isogenic ftsZ, ftsQ, ftsA, pbpB, and ftsE cell division mutants of Escherichia coli during temperature shift experiments. J Bacteriol 170:1533–40.

3. Datsenko KA, Wanner BL. 2000. One-step inactivation of chromosomal genes in Escherichia coli K-12 using PCR products. Proc Natl Acad Sci 97:6640–6645.

4. Blattner FR, Plunkett G, Bloch CA, Perna NT, Burland V, Riley M, Collado-Vides J, Glasner JD, Rode CK, Mayhew GF, Gregor J, Davis NW, Kirkpatrick HA, Goeden MA, Rose DJ, Mau B, Shao Y. 1997. The complete genome sequence of Escherichia coli K-12. Science 277:1453–62.

5. Denome SA, Elf PK, Henderson TA, Nelson DE, Young KD. 1999. Escherichia coli mutants lacking all possible combinations of eight penicillin binding proteins: Viability, characteristics, and implications for peptidoglycan synthesis. J Bacteriol 181:3981–3993.

6. Den Blaauwen T, Aarsman MEG, Vischer NOE, Nanninga N. 2003. Penicillin-binding protein PBP2 of Escherichia coli localizes preferentially in the lateral wall and at mid-cell in comparison with the old cell pole. Mol Microbiol 47:539–47.

7. Alexeeva S, Gadella TWJ, Verheul J, Verhoeven GS, den Blaauwen T. 2010. Direct interactions of early and late assembling division proteins in Escherichia coli cells resolved by FRET. Mol Microbiol 77:384–98.

8. Verhoeven GS, Dogterom M, den Blaauwen T. 2013. Absence of long-range diffusion of OmpA in E. coli is not caused by its peptidoglycan binding domain. BMC Microbiol 13:66.
